# Social isolation induces sexually aggressive behaviour in male Wistar rats

**DOI:** 10.1186/s12868-025-00932-0

**Published:** 2025-02-26

**Authors:** Mbiydzenyuy Elvis Ngala, Sian Megan Joanna Hemmings, Jacqueline Samantha Womersley, Thando W. Shabangu, Lihle Qulu-Appiah

**Affiliations:** 1 Division of Medical Physiology, Biomedical Science Research Institute, Stellenbosch University, Private Bag X1, Matieland, Cape Town, 7602 South Africa; 2 Department of Psychiatry, Faculty of Medicine and Health Sciences, Stellenbosch University, Cape Town, 7505 South Africa; 3 SAMRC/Stellenbosch University Genomics of Brain Disorders Research Unit, Faculty of Medicine and Health Sciences, Stellenbosch University, Cape Town, ZA 7505 South Africa

**Keywords:** Sexual violence, Wistar rats, Sexual aggression, Social isolation, HPA axis, Neurochemical markers, Gene expression, Oxytocin, Vasopressin

## Abstract

**Background:**

Sexual violence, a pervasive global issue, significantly impacts individuals and societies, necessitating a deeper understanding of its underlying biological mechanisms. This study aimed to elucidate the role of stress-induced dysregulation of the hypothalamus-pituitary-adrenocortical axis in sexual aggression in male Wistar rats. Employing a sexual aggression paradigm, we investigated the effects of social isolation on aggression, anxiety-like behaviour, and neurochemistry in virgin adult male Wistar rats.

**Results:**

The results showed that social isolation significantly escalated aggressive behaviours and induced anxiety-like responses in male rats. The sexual aggression test revealed that socially isolated males exhibited heightened aggression towards non-receptive females. Neurochemical analyses indicated significant alterations in key markers, such as corticotrophin-releasing hormone, oxytocin, and arginine vasopressin, correlating with the observed behavioural changes. Gene expression analyses revealed significant findings, particularly in the expression of the oxytocin receptor (OXTR) and vasopressin receptor 1 A (AVPR1A) genes. Social isolation and the duration of aggressive behaviour prior to the sexual aggression test significantly influenced *OXTR* expression in the hippocampus and *AVPR1A* expression in both the prefrontal cortex and hippocampus, highlighting the complex interplay between environmental stressors, neurochemical responses, and gene expression in the manifestation of sexual aggression behaviour.

**Conclusions:**

This study underscores the critical impact of stress and social isolation on sexual aggression, providing valuable insights into possible neurobiological underpinnings of sexual violence. Understanding these mechanisms is crucial for developing effective interventions to mitigate the consequences of sexual aggression.

**Supplementary Information:**

The online version contains supplementary material available at 10.1186/s12868-025-00932-0.

## Introduction

Sexual violence, encompassing a spectrum of coercive acts such as sexual assault and harassment, constitutes a critical global issue with profound impacts across all demographics. The World Health Organization estimates that nearly one-third of women globally have encountered some form of sexual violence, highlighting the issue’s ubiquity [1]. This challenge is compounded in regions recently affected by military conflicts, where a culture of militarised masculinity may exacerbate the normalisation of aggressive behaviours, including sexual aggression [2, 3].

In the animal kingdom, aggression is integral to social dynamics and resource competition, serving adaptive functions in survival and hierarchy formation [4]. However, it poses significant risks, including psychological trauma and increased mortality [5]. The complexity of sexual aggression behaviour and its potential exacerbation by stress factors necessitate a comprehensive understanding of its underlying mechanisms and modulating biological factors to devise effective prevention and intervention strategies [6].

Stress is a significant disruptor of homeostasis. It activates a cascade of structural, biochemical, and physiological responses aimed at re-establishing equilibrium and ensuring survival [7]. Key brain regions such as the medial prefrontal cortex, hippocampus, and amygdala, which are integral to learning, memory, emotional processing, and cognitive functions, undergo substantial remodelling under stress. This remodelling significantly impacts the regulation of the hypothalamus-pituitary-adrenocortical (HPA) axis, a pivotal stress response system. The glucocorticoids released by the adrenal glands as the end products of HPA axis activation orchestrate the physiological and behavioural responses to stress [8]. Chronic stress, however, can lead to HPA axis dysregulation, preventing the restoration of homeostasis and potentially resulting in behavioural alterations such as anxiety, depression, and aggression [9, 10].

Several studies have shed light on the complex interplay between stress, neuromodulators, and aggressive behaviour. For instance, research by Raise-Abdullahi et al., (2023) demonstrated that chronic stress leads to alterations in the expression of corticotropin releasing hormone (CRH) receptors in the brain, thereby influencing stress responses and social behaviours, including aggression [11]. Investigations by Inoue et al., (2023) revealed that testosterone levels are positively correlated with aggressive behaviours in male mammals, highlighting the role of this hormone in regulating territorial and dominance-related aggression [12]. Similarly, serotonin deficiency has been associated with increased aggression levels in animal models, underscoring the significance of serotonin in modulating mood and impulse control [13]. Research shows that the influence of vasopressin on aggression depends on sex. For example, Fodor et al., (2014) found that vasopressin deficiency affects aggression and impulsiveness differently in male and female rats, suggesting that vasopressin’s effects on maternal aggression develop alongside impulsivity [14]. Stribley and Cater (1999) demonstrated that postnatal exposure to arginine vasopressin (AVP) leads to long-lasting increases in aggression in adult male prairie voles [15]. Early foundational research by Feris et al., (1989) highlighted that vasopressin levels in the anterior hypothalamus fluctuate with the oestrous cycle, indicating a complex interplay between hormonal cycles and aggressive behaviour [16]. Additionally, Delville et al., (1998) provided evidence that vasopressin innervation in the brain is associated with flank-marking behaviour in golden hamsters, further underscoring vasopressin’s role in aggression [17]. Oxytocin also has complex effects on aggression. Insel and Winslow (1991) illustrated the effects of central oxytocin administration on social behaviours, including responses to social separation in infant rats, laying the groundwork for later studies on oxytocin’s role in social and aggressive behaviours [18]. Moreover, Pfundmair et al., (2018) found that oxytocin promotes aggression in response to provocation in low-anxiety individuals [19]. Similarly, DeWall et al., (2014) showed that oxytocin increases intimate partner violence inclinations in individuals prone to physical aggression [20]. Conversely, Gulevich et al., (2019) reported that a single oxytocin application decreases the time spent engaged in aggression and increases the latency period to this behaviour in aggressive rats but had no such effect in tame rats [21]. These findings emphasise the importance of investigating the neurobiological substrates underlying aggressive behaviours to gain a comprehensive understanding of their mechanisms and potential therapeutic targets.

Despite the critical need to understand the determinants of male sexual aggression, research in this area, particularly in animal models, remains sparse. Existing human studies focus on the psychological profile of offenders, with limited exploration of physiological underpinnings [22, 23]. Such studies are also often hampered by methodological limitations such as confirmation bias [24]. Within the context of aggression research, various animal models have been developed to elucidate the multifaceted nature of aggressive behaviours, each with its own specific focus and limitations. Though these cannot fully replicate the multifaceted and complex nature of aggression in humans, their use to explore the neurobiology underlying sexual aggression is particularly valuable. Traditional models of aggression, such as the resident-intruder paradigm, have been instrumental in studying territorial and dominance-related aggression often employing same-sex animals to assess aggressive interactions. This paradigm involves introducing an “intruder” animal into the territory of a “resident” animal and observing the ensuing interactions. The Sexual Conspecific Aggressive Response model offers valuable insights into the neurochemical and structural changes that may underlie sexual aggression [25]. However, this model primarily addresses the impact of aggressive sexual behaviour on female rats, focusing on the neurochemical and structural changes associated with sexual aggression, with scant information on the biological mechanisms driving the development of sexual aggression in males. While these models offer valuable insights into mechanisms underlying general aggression, they fall short in accurately depicting the complexities of sexual aggression, which intertwines sexual motivation with aggressive behaviour. The Sexual Aggression Test (SxAT) has emerged as a specialised model designed to specifically address male sexual aggression [26]. The SxAT aims to replicate and measure sexually aggressive behaviours under controlled laboratory conditions, providing a structured framework to assess the interplay between sexual arousal and aggression.

The current study seeks to establish an adapted version of the SxAT based on the original methodology developed by Oliveira et al., (2022) [26]. By subjecting male Wistar rats to seven days of social isolation to induce stress and HPA axis dysregulation, and using the resident-intruder test to assess aggression, we endeavoured to simulate conditions conducive to the development of aggressive behaviour. Subsequently, a four-day sexual aggression assessment protocol was implemented to further investigate the sexual aggressive responses elicited by these conditions. This adapted animal SxAT model aims to better investigate the specific dynamics and determinants of sexually aggressive behaviours in male Wistar rats in the context of social isolation.

## Materials and methods

### Animals

The study used virgin adult male (250–300 g) and female (180–200 g) Wistar rats, *Rattus norvegicus*, obtained from the Stellenbosch University Animal Breeding Facility. The animals were housed in type III cages, maintaining a controlled environment with a room temperature of 22 ± 1 °C, 55% relative humidity, and an inverted 12-hour light/dark cycle (lights off at 06:00, lights on at 18:00). To address the impact of environmental enrichment on social dynamics, enrichment materials were introduced to the cages of group-housed males and all females. As environmental enrichment has been shown to mitigate the effects of isolation, cages for socially isolated male rats remained unenriched [27]. Socially isolated males were housed individually, while group-housed males were accommodated in groups of 3–4 per cage. Both food and water were provided *ad libitum*.

Animal care and experimental procedures were conducted in strict compliance with the South African National Health Research Ethics guidelines and were approved by the Stellenbosch University Research Ethics Committee (ACU-2021-13333). Prior to the commencement of experimental protocols, all animals underwent a seven-day habituation period within the home room to acclimate to the environment, as well as handling. Following habituation, the rats were divided into groups (*n* = 10 per group) as detailed below:

### Adult male rats

Group I (GHCM): Group housed control males. This group served as the control. Males were housed in groups and were not exposed to the SxAT.

Group II (GHSM): Group housed sexual aggression assessment males. Males in this group were housed in groups to study sexual aggression in a social housing context.

Group III (ICM): Isolated control males. Males were isolated to assess the impact of social isolation without exposure to the SxAT.

Group IV (ISM): Isolated sexual aggression assessment males. Males were isolated to study sexual aggression in the context of social isolation.

Group V: Intruders (*n* = 5): These were lighter, younger, physically smaller and well-socialised male rats that were never housed under isolated conditions. The rats were used in the resident intruder paradigm and were unfamiliar to the resident rats to ensure clear differentiation and to minimise pre-existing social hierarchies that could influence the outcome of the test.

Group VI: Sexually experienced males (*n* = 5). These males were not enrolled in behavioural experiments but were used to ensure reliable mating behaviour during the assessment of female sexual receptivity.

### Adult female rats

Group I (CF): Control females never exposed to males.

Group II (GHSF): Females exposed to group housed males during the SxAT.

Group III (ISF): Females exposed to isolated males during the SxAT.

**Fig. 1:**
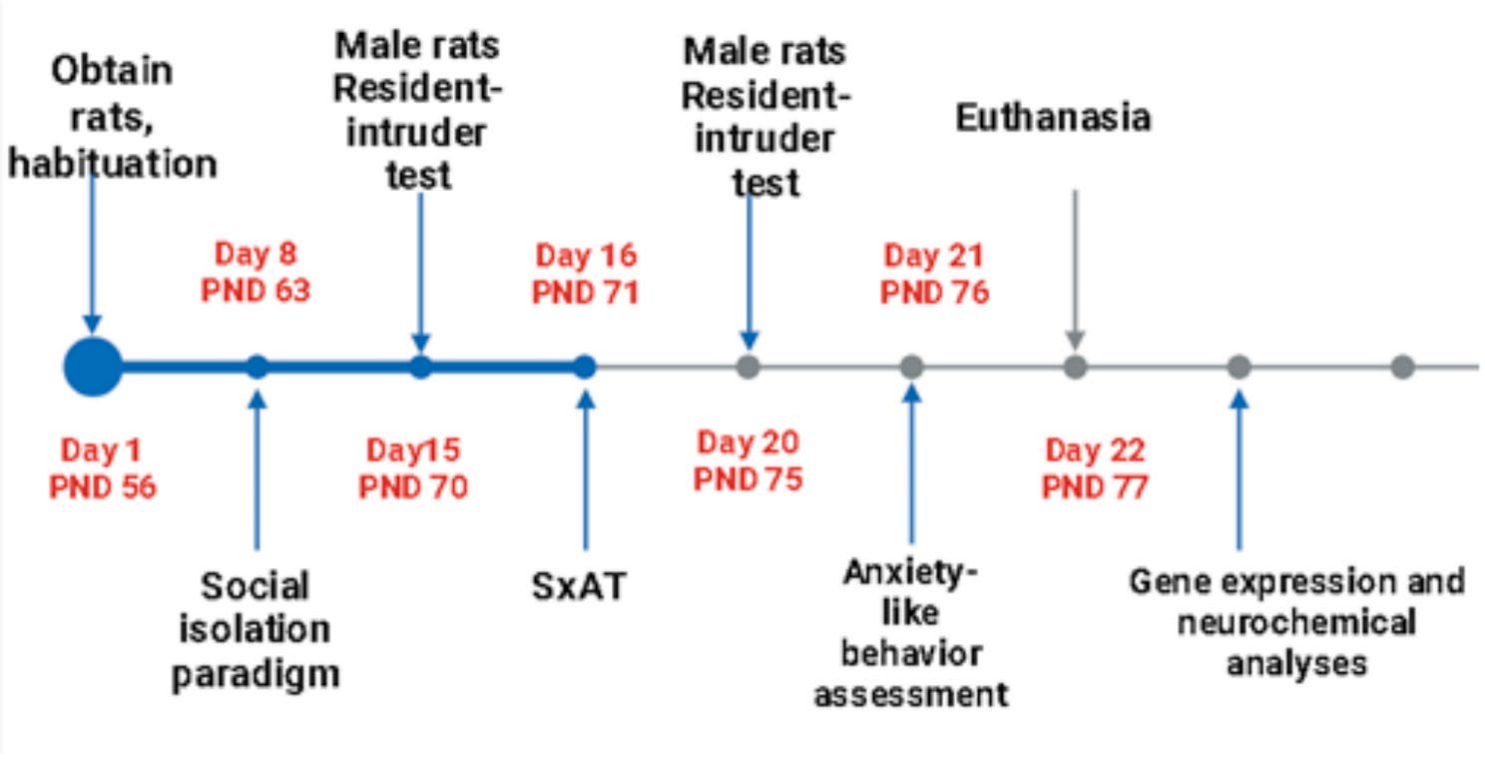
Timeline of study design and behavioural testing procedures. This timeline illustrates the sequence of key events and procedures in the study, including time points for acclimation, social isolation, the order of behavioural tests, and sample collection. Each step is depicted to provide a clear understanding of the study’s methodological flow and ensure reproducibility. SxAT = sexual aggression test

The study proceeded as indicated in the timeline (Fig. 1).

### Induction of stress (social isolation paradigm)

To induce stress through social deprivation, male rats at postnatal day (PND) 63 (9 weeks old) were housed individually in single cages for seven days, as previously described [28]. Rats assigned to the social condition were grouped and housed 3–4 per cage, facilitating normal social interactions among them. Both isolated and social groups were maintained in the same room, ensuring uniform environmental conditions. Interactions with the experimenter were minimised and restricted solely to essential handling during the weekly maintenance of the cages, thus maintaining the integrity of the social isolation conditions for the isolated group.

### Resident-intruder paradigm

The resident-intruder paradigm was used to evaluate whether social isolation elicited aggressive behaviour in male rats. This was performed on the day before the SxAT (GHCM, ICM, GHSM and ISM) and then again on the day after the SxAT (for ISM and GHSM rats). The assessments were scheduled at 13:00 during the dark phase, under dim red illumination (< 2 lx) to facilitate video recording while preserving the nocturnal conditions conducive to rat activity. To enhance territorial behaviour, the bedding in the home cages of both isolated and group-housed males remained unchanged for three days leading up to the test.

The resident-intruder test was conducted on male rats as previously described [29]. On the day of the experiment, (PND 70), the weights of the experimental males (residents) and the intruders were recorded to ensure that intruders were lighter [30, 31]. At 12:00, residents were moved to transparent cages with their original bedding for a one-hour acclimation period in their home environment. Following that, both residents and intruders were relocated to the experimental room for an additional hour of habituation. A 15-minute pre-test period was allotted for the animals to adjust to the presence of the experimenter, red light, and camera setup, minimising the confounding effects of the novel environment on behaviour.

A male intruder was introduced into the home cage of the resident, during which dominant and subordinate behaviours, latency to the first attack, and the frequency of attacks by the resident were observed and recorded over a 10-minute duration. Behavioural analyses were performed by an observer blinded to the experiment using Behavioural Observation Research Interactive Software (BORIS) [32] and focussed on three main categories: (i) aggressive behaviours, including keep down, threat behaviour, offensive grooming, and attacks; (ii) neutral behaviours, such as exploration, and self-grooming; and (iii) social behaviours, encompassing non-aggressive interactions like sniffing [31].

### Assessment of sexual aggression behaviour

To assess if the aggression elicited by social isolation provoked sexual aggression in male rats towards non-receptive female rats, we performed the SxAT from PND 71. Prior to the test each day, the female rats were screened for receptivity.

### Identification of sexually receptive and non-receptive female rats

To differentiate between sexually receptive and non-receptive female rats, daily assessments of the oestrous cycle were conducted using vaginal cytology, which is crucial for understanding reproductive behaviour and physiology [33, 34]. The oestrous cycle comprises four distinct stages: proestrus, characterised by growing ovarian follicles and round nucleated epithelial cells; oestrus, denoted by sexual receptivity and the predominance of large cornified cells; metestrus, marked by numerous corpora lutea and leukocytes; and dioestrus, indicated by the presence of small follicles and a predominance of leukocytes [35].

Vaginal smears were collected at 09:00 using a cotton bud moistened with saline, introduced approximately 1 cm into the vaginal orifice to avoid cervical stimulation that could induce pseudopregnancy [36]. The epithelial cell samples were air-dried, mounted on slides, and examined under a microscope at 10x and 40x magnifications without staining. This process enabled the identification of the oestrous stage based on the cell composition in the vaginal smears.

For experimental purposes, the oestrous cycle stages were categorised into two groups: “oestrous” for females in proestrus or oestrus stages, indicating sexual receptivity, and “non-oestrous” for those in metestrus or dioestrus, denoting a lack of receptivity [35, 37]. This classification is pivotal in studies exploring sexual aggression and receptivity in rodents and was thus performed prior to subsequent behavioural analyses.

### Assessment of receptivity

To evaluate sexual receptivity among female rats identified as being in the oestrous phase, a structured behavioural assay was implemented at a consistent daily time of 11:00 AM, following oestrous confirmation via vaginal cytology. This timing ensured hormonal alignment conducive to sexual receptivity. To assess for receptivity, a female rat in oestrous was paired with a sexually experienced male rat, not part of the experimental cohort, to observe and record receptive behaviours indicative of readiness to mate. Key behaviours monitored included lordosis, characterised by an arched back posture to facilitate male intromission; darting and hopping movements signifying arousal; and ear-wiggling, a less common but recognised indicator of female receptivity [38].

Upon demonstration of these behaviours, the female was immediately removed to prevent mating, ensuring the focus remained on receptivity rather than copulation. This procedure was repeated over four consecutive days to systematically assess the persistence and escalation of sexually aggressive behaviours following repeated exposures [39], thereby providing a comprehensive assessment of the females’ readiness to engage in mating behaviours across distinct stages of their hormonal cycle.

### Sexual aggression test protocol

We employed an adapted version of the SxAT [26]. Our adaptations included:

1. Housing conditions: We specifically modified the housing conditions prior to the SxAT to include both isolated and group-housed settings. This allowed us to assess the impact of the social environment on aggressive behaviours.
2. Aggression induction: To induce aggressive behaviour, we used a resident-intruder paradigm where male rats were exposed to intruder male rats before the SxAT. This step was designed to enhance the ecological validity of the test by simulating a competitive social scenario that could trigger aggressive responses.
3. Neurochemical and gene expression analysis: In addition to behavioural observations, we incorporated neurochemical and gene expression analyses to explore the underlying mechanisms of aggression. We measured levels of key markers and gene expression in specific brain regions.

The core principles of the original SxAT methodology were maintained, including the interaction between male and non-receptive female rats to induce and measure sexual aggression behaviours.

The test was conducted in a controlled environment, specifically in a dark phase illuminated by very dim red light (less than 2 lx), to facilitate video recording while preserving the nocturnal conditions conducive to rat activity.

On PND 71, after screening the female rats at 09:00 to determine the oestrous status, and assessing for sexual receptivity at 11:00, both male (GHSM and ISM) and female (GHSF and ISF) rats were assessed using the SxAT as briefly described below.

At 12:00, the experimental males (GHSM and ISM) and females (GHSF and ISF), were moved to filming cages with their home cage bedding and allowed to habituate for 1 h in their home room.

At 13:00, a sexually receptive female in oestrus was placed in a cage with an experimental male. After successful intromission, the female was immediately replaced by a non-receptive female that was not in oestrus. Male-female pairings were recorded for the remaining time within the 10-minute recording period.

Sexual aggression behaviour was indicated by the male rat forcefully mounting, as assessed by placement of the forepaws and pelvic thrusting, an unreceptive female rat [40]. Female lack of receptivity was indicated by a non-lordotic posture, kicking back, lying down on the back, or turning around upright. The male sexual behaviour was recorded alongside other aggressive behaviours, which included: forced grooming (male rats aggressively licking the head/neck area of the female), keep down (males using their front paws and upper body to force the female to lie on her back), threat (males displaying threatening postures or movements toward the female, including pushing or shoving her with their head), and lateral threat (males turning their bodies sideways and pushing the female into a wall or corner).

A trained observer used a timer to record the copulation latency, as well as the occurrence of forced mounting and aggressive behaviour. This produced a frequency score (number of behaviours in 10 min) for forced mounting and aggressive behaviour. Additionally, every SxAT were captured on camera using a GoPro (Sumikon, PEARL GmbH, Buggingen, Germany). Therefore, in addition to forced mounting and aggressive behaviour, neutral behaviours (such as immobility, exploration, and self-grooming) and non-aggressive social interactions (such as anogenital sniffing and defensive behaviour) were also recorded and graded. The percentage of time an animal spent engaging in a particular type of behaviour was determined from these scores (total length of behaviour / 600 × 100%).

Control groups, including GHCM, ICM, and CF, were not exposed to rats of the opposite-sex but underwent the same recording procedures in the experimental room, ensuring consistency across all experimental conditions.

After the SxAT, the male rats (GHSM AND ISM) underwent evaluations for aggressive behaviour (on PND 75) through the previously described resident-intruder test, alongside assessments for anxiety-like behaviour using both the open field test (OFT) and elevated plus maze (EPM).

### Assessments of anxiety-like behaviour

On PND 76, male rats were assessed for anxiety-like behaviour using the EPM and OFT, according to previously described methods [41, 42]. These evaluations were scheduled 48 h following the conclusion of the SxAT to ensure any immediate stress responses had subsided. At 12:00, prior to testing, the rats were transferred to a holding room, where they were given a one-hour period to acclimate to the unfamiliar environment. The testing procedures commenced at 13:00, beginning with the EPM and followed by the OFT, to systematically assess the rat’s anxiety-like behaviour under controlled conditions. To ensure even illumination, testing chamber cages were not placed in direct light, dark corners, or shadowed areas. All instrument acclimation and testing were performed at the same time each day between the hours of 12:00 and 17:00 and by the same individuals, who were blinded to the animal treatment group.

### The open field test

The OFT was used to assess anxiety-like behaviour. This 72 × 72 × 36 cm apparatus is divided by a painted line into central (measuring 24 × 24 cm) and outer zones. Male rats were placed into the cleaned apparatus and behaviour was recorded for 5 min using an overhead camera. Horizontal (ambulation and freezing) and vertical movements were registered and used as measures of locomotor activity, exploration and anxiety-like behaviour. Grooming and freezing were used as measures of anxiety [43]. The apparatus was cleaned with a 70% alcohol solution and allowed to dry between each test. Behaviour was analysed using the BORIS software. The paradigm relies on the approach-avoidance conflict, in which the drive to explore novel environments is in opposition to the tendency of prey animals, such as rats, to avoid open spaces. Time spent in the arena’s periphery serves as a robust marker of anxiety-like behaviour, is responsive to anxiolytic interventions, and is used in models of stress-induced anxiety conditions [44].

### The elevated plus maze

The EPM assay was performed as previously described [42]. Briefly, the maze consisted of two closed arms (50 × 10 × 40 cm) and two open arms (50 × 10 cm), which were elevated 50 cm above the floor. The intersection between the open arm and the closed arm was an open square platform (10 × 10 cm). The rats were placed individually on the central platform facing a closed arm and allowed to explore the maze for 5 min. Their behaviour was monitored using a video camera and analysed with BORIS software. The percent time spent and the number (frequency) of entries into the open arms were recorded. The entire apparatus was cleaned with a 70% alcohol solution and dried properly between each test.

### Brain tissue preparation

To allow for stabilisation of hormone levels and mRNA expression, all animals were sacrificed by decapitation using a sharp guillotine 24 h after the last behavioural test (PND 77). Brain tissue (amygdala, hippocampus, hypothalamus, and prefrontal cortex) was harvested, snap-frozen in liquid nitrogen and stored at -80 °C in a Bio-freezer for later analysis of mRNA and gene expression differences.

### Neurochemical assessments

Trunk blood samples from male rats (*n* = 10 per group) were collected into serum collection tubes and centrifuged at 1200 rpm for 20 min at 4 ^o^C using a Heraeus Labofuge 200 centrifuge (Thermo Fisher Scientific, Waltham, MA). The serum supernatant was then aspirated with a 1000 µl pipette, transferred into 1 ml Eppendorf tubes, and stored at -80 °C for later analysis. Serum samples were subsequently thawed and the concentration of neurochemicals was determined according to the manufacturer’s instructions using enzyme-linked immunosorbent assay (ELISA) kits (Elabscience, Houston, TX). This assay involves precoating microplate wells with antibodies specific to the target neurochemical. The neurochemical present in the samples competes with a fixed amount of neurochemical-horseradish peroxidase (HRP) conjugate for binding sites on the antibody. After incubation, unbound conjugates are removed through a series of wash steps, resulting in an inverse relationship between the amount of bound neurochemical-HRP conjugate and the concentration of neurochemical in the sample. The development of the colorimetric signal, initiated by adding a substrate solution, is inversely proportional to the neurochemical concentration, measured by optical density at 450 nm (FLUOstar Omega, BMG LABTECH, Ortenberg, Germany). The serum concentrations of testosterone (Elabscience^®^ QuicKey Pro Rat T(Testosterone) ELISA Kit- E-OSEL-R0003), oxytocin (Elabscience^®^ QuicKey Pro Rat OT(Oxyctocin) ELISA Kit- E-EL-0029), corticosterone (Elabscience^®^ QuicKey Pro Rat CORT(Corticosterone) ELISA Kit- E-EL-0160), CRH (Elabscience^®^ QuicKey Pro Rat CRH (Corticotropin Releasing Hormone) ELISA Kit- E-EL-R0270), and serotonin (Elabscience^®^ QuicKey Pro Rat 5-HT (5-Hydroxytryptamine) ELISA Kit- E-EL-0033), were quantified using rat-specific ELISA kits. Given the low concentration of AVP in serum and the potential for interference from other serum components, an extraction procedure was performed prior to immunoassay analysis. The extraction involved mixing 1 part serum sample with 1.5 parts of Extraction Solution, vortexing, and then incubating samples at room temperature for 90 min. The mixture was then centrifuged at 4 °C at 1660 x g for 20 min. The supernatant was transferred to a clean tube and dried using a speedvac (Thermo Fisher Scientific) at 37 °C. The samples were then reconstituted with 250 µL of Assay Buffer. Following extraction, the serum levels of AVP were quantified using a highly sensitive and specific immunoassay kit for AVP (DetectX^®^ Arg8-Vasopressin (AVP) Immunoassay Kit, ARBOR Assays-K049-H), according to the manufacturer’s protocol. The analyses of the samples were carried out in triplicates, and the values were estimated from the standard curve produced from the calculated concentrations.

### RNA extraction, cDNA synthesis, and quantitative real-time PCR

The stored brain tissue was homogenised using the lysis buffer included in the RNA isolation kit (Cat No. 83913-1EA) provided by Sigma-Aldrich (St. Louis, MO, USA). Total RNA was extracted using the GenElute™ Total RNA Purification Kit (Sigma-Aldrich). RNA purity was determined using the Nanodrop spectrophotometer (NANODROP ONE, Thermofisher Scientific, Madision, WI, USA) by measuring the ratio of absorbance at 260 nm to 280 nm (A260/A280 ratio). A ratio between 1.8 and 2.0 was considered indicative of pure RNA, free from protein contamination. RNA integrity was assessed via gel electrophoresis on a 1% agarose gel. The appearance of sharp 28 S and 18 S ribosomal RNA bands confirmed the integrity of the RNA, with minimal smearing indicating little to no degradation [45]. RNA quality was measured using TapeStation (RNA ScreenTape). An RNA Integrity Number (RIN), above 7 (scale from 1 to 10 used to assess RNA quality, with 10 indicating intact RNA) was considered acceptable for sensitive downstream applications such as cDNA synthesis and quantitative real-time PCR (qPCR), ensuring reliable and accurate gene expression analysis.

The extracted RNA samples were reverse-transcribed at 37 °C for 120 min using the High-Capacity cDNA Reverse Transcription Kit 4,368,814 (Thermo Fisher Scientific) according to the manufacturer’s protocol. cDNA derived from 10 ng of RNA was used for qPCR under the following conditions: 10 min at 95 °C, 40 cycles of 15 s at 95 °C, and 1 min at 60 °C using 200 nM forward and reverse primers. In the qPCR analysis, pre-designed primers targeting the genes of interest (AVPR1A, CRHR1, OXTR, AR, and Htr1a, Supplementary Table 1) were obtained from Thermo Fisher Scientific. The qPCR assays were performed under standard conditions using a real-time PCR system (Quant studio 5) and the TaqMan^®^ Fast Advanced Master Mix kit (Thermo Fisher Scientific) following the manufacturer’s protocol. Each assay was conducted in triplicate to ensure robustness and reliability of the results.

To determine the amplification efficiency of each assay and to calculate relative expression levels, standard curves were prepared using serially diluted cDNA samples (gene amplification efficiencies; *AVPR1A* = 106.382, *GAPDH* = 93.386, *HTR1A* = 84.276, *OXTR* = 89.487, *AR* = 90.123, *CRHR1* = 86.345). The standard curves were generated using a range of known concentrations of cDNA, enabling us to assess the efficiency of amplification and establish the relationship between Ct values and the quantity of target mRNA.

The Ct values obtained from the qPCR experiments were adjusted using the following formula: Adjusted Ct (Ct_adj) = Ct_sample + [(Ct_standard - Ct_intercept) / slope]. This adjustment accounts for variations in amplification efficiency and ensured accurate quantification of gene expression levels across samples.

Glyceraldehyde 3-phosphate dehydrogenase (GAPDH) was chosen as the endogenous control due to its stable expression across various tissues and experimental conditions [46]. GAPDH is widely utilised as a reference gene in qPCR experiments owing to its involvement in glycolysis and its constitutive expression, making it a reliable indicator for mRNA quantity and quality normalisation.

Samples from various experimental groups and brain regions were randomly distributed across qPCR plates to ensure impartial and representative outcomes. This random allocation minimises bias and ensures that observed differences reflect genuine biological variations. Randomisation also addresses plate-to-plate variability and evens out systematic effects, enhancing the reliability of our qPCR data analysis.

The comparative Ct or ΔΔCt method was used to calculate the relative fold change (2^−ΔΔCt^) in gene expression from qPCR experiments.

ΔCt_sample_​=Ct_Target gene, sample_​− Ct_Housekeeping gene, sample_​.

ΔCt_calibrator_​=Ct_Target gene, control_​− Ct_Housekeeping gene, control_.

ΔΔCt_sample​_ = ΔCt_sample​_ − ΔCt_calibrator​_.

Relative Fold Change = 2^−ΔΔCtsample^.

### Data and statistical analysis

The R programming language, designed for statistical computing and graphics, was used for statistical analysis [47]. The distribution of the behavioural assessment data was checked using the Shapiro-Wilk test. Outliers were identified using Grubb’s test and removed. For normally distributed data, comparisons between two groups were performed using Welch two-sample t-tests. Data are presented as the mean ± SD. For non-normally distributed data, the median values between groups were compared using the Kruskal-Wallis Rank Sum test, and data are presented as the mean and interquartile range (IQR). Regression analyses were used to assess group differences in aggressive behaviour during the resident intruder paradigm tests conducted after exposure to the SxAT. Regression models included post SxAT behaviour as the outcome variable and social isolation, pre SxAT behaviour, and the social isolation x pre SxAT behaviour as predictors. Linear mixed models to assess differences in SxAT aggressive behaviours across the four days of testing used test day and social isolation groups as fixed effects and the rat ID as a random effect. Mixed models were run using the *lme4* package [48], and p-values were generated using Kenward-Roger approximation of degrees of freedom in the *lmertest* package [49]. Significant outcomes were probed using the lsmeans function with Tukey correction. Correlations between behavioural and neurochemical measures were assessed using Pearson’s correlation coefficient for normally distributed data and Spearman’s Rho for data that were not normally distributed. Significance was set at *p* < 0.05.

## Results

### Housing in isolation increased aggressive behaviour in the resident-intruder test before the SxAT

**Fig. 2:**
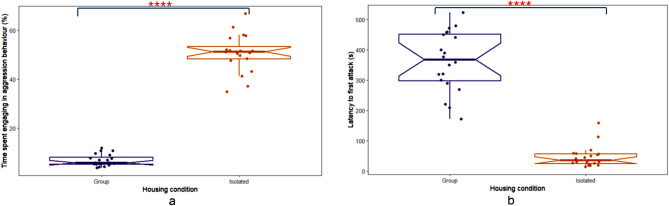
Housing in isolation increased aggression in the pre-SxAT resident intruder test. Isolated Rats housed in isolation (‘Isolated’, *n* = 20) (**a**) spent more time exhibiting aggressive behaviour and (**b**) had a shorter latency to first attack those reared in groups (‘Group’, *n* = 20) during the 10-minute test. Raw data points are plotted, with box plots representing the median, interquartile range, whiskers for values 1.5x the interquartile range, and notches for the 95% confidence interval. **** *p* < 0.0001

To investigate baseline aggressive tendencies in male Wistar rats subjected to social isolation prior to the SxAT, aggressive behaviours between rats in the isolated (ICM & ISM) and group-housed (GHCM & GHSM) conditions using the resident-intruder test were compared using t-tests or Wilcoxon-Rank sum tests, as appropriate (Table 1). Social isolation significantly influenced aggressive behaviour in the resident-intruder test, with isolated rats (ICM & ISM) spending a significantly higher percentage of time engaged in aggressive behaviour (50.73 ± 5.05%) compared to group-housed rats (GHCM & GHSM) (6.87 ± 2.83%) during the pre-SxAT period (W = 0, *p* = 0.0000) (Fig. 2a). Housing condition significantly affected the latency to the first attack in the pre-SxAT period, with isolated rats (ICM & ISM) exhibiting a significantly shorter latency (36.5 ± 32 s) compared to group-housed rats (GHCM & GHSM) (369.0 ± 153.75 s) (W = 400, *p* = 0.0000) (Fig. 2b).

**Table 1:** Impact of isolated housing on resident intruder test aggressive behaviours in rats prior to the SxAT

Behavioural parameter (s)	Group-housed median (IQR)	Housed in isolation median (IQR)	Test statistic value	*P*-value
Forced mounting duration ^@^	2.5 (2.0–3.3)	18.0 (15.8–24.3)	W = 0	0.0000***
Threats duration ^@^	4.0 (3.0–5.3)	26.0 (14.5–32.3)	W = 0	0.0000***
Keep down duration ^@^	5.0 (3.8–8.0)	68.5 (47.5–81.0)	W = 0	0.0001***
Offensive grooming duration ^@^	6.5 (3.8–15.3)	41.0 (31.8–49.8)	W = 6	0.0002***
Attack duration ^#^	19.1 ± 5.2	154.8 ± 31.1	t = -19.255	0.0000***
Latency to attack ^@^	369.0 (298.3–452.0)	36.5 (25.3–57.3)	W = 0	0.0000***

This table presents the aggression behavioural parameters of rats subjected to different housing conditions (‘Group’ vs. ‘Isolated’) before undergoing the SxAT. ^#^ Parametric data are reported as the mean and standard deviation and were compared using the Welch two sample t-test. ^@^ Non-parametric data are reported as the median and 25th and 75th percentile values and were compared using the Wilcoxon rank sum test. Statistically significant differences were observed across all parameters (*p-values < 0.0001). Sample size for each group is n = 20.

### Housing in isolation increased aggressive behaviour in the resident-intruder test after the SxAT

**Fig. 3:**
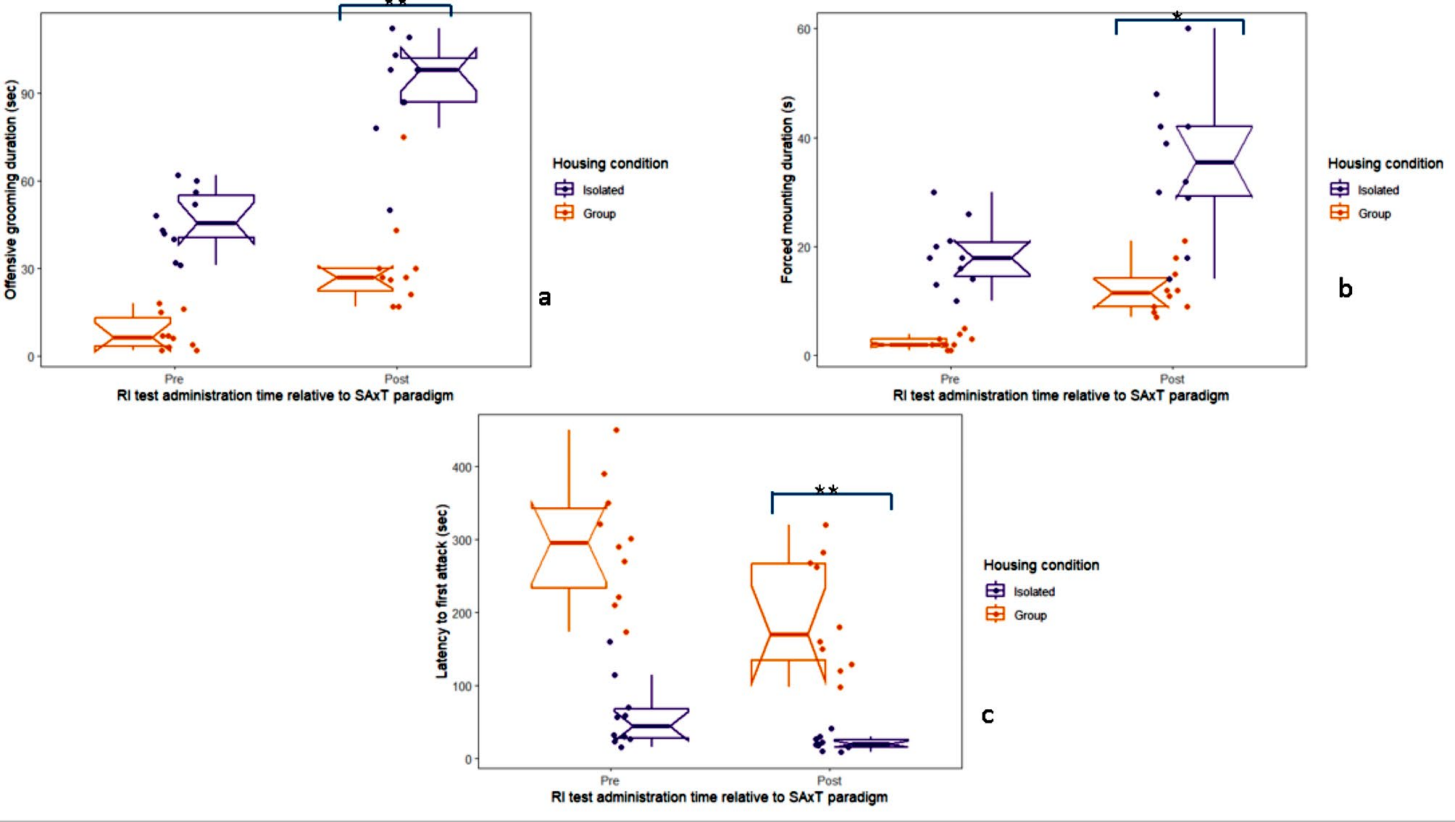
Housing in isolation increased aggression in the post-SxAT resident intruder test. The analyses compared isolated (ISM, *n* = 10) and group-housed (GHSM, *n* = 10) rats, considering pre-SxAT behaviour as a predictor. Isolation housing significantly increased the duration of (**a**) offensive grooming and (**b**) forced mounting and (**c**) reduced the latency to first attack. No significant interaction effects between housing condition and pre-SxAT vs. post-SxAT behaviours were observed. Raw data points are plotted, with box plots representing the median, interquartile range, whiskers for values 1.5x the interquartile range, and notches for the 95% confidence interval. *p < 0.05, **p < 0.01

Regression analyses were employed to evaluate the impact of housing conditions on aggressive behaviour in the resident intruder paradigm conducted after the SxAT. Models employed pre-SxAT behaviour and housing condition (ISM vs. GHSM), as well as their interaction, as predictors with aggressive behaviours as outcomes. The analysis highlighted a significant main effect of housing condition on three behaviours. Isolation housing was associated with higher “offensive grooming” (beta estimate = 88.970, SE = 26.699, t = 3.332, *p* = 0.0042) (Fig. 3a) and “forced mounting” (beta estimate = 34.355, standard error = 13.817, t = 2.486, *p* = 0.0243) (Fig. 3b) durations, and shorter latency to first attack (beta estimate = -248.079, standard error = 75.477, t = -3.287, *p* = 0.0047) (Fig. 3c). The latency to attack regression model explained a substantial portion of the variance in latency to first attack (adjusted R-squared = 0.7062). No models showed housing condition x pre-SxAT interaction effects (Table 2).

**Table 2:** Impact of isolation housing on aggressive behaviour post-SxAT in male Wistar rats

Behavioural parameter (s)	Model coefficients	Estimate	Standard error	t value	Pr(>|t|)
Forced mounting duration	Isolated housing condition	34.355	13.817	2.486	0.0243*
	Pre-SxAT forced mounting duration	0.966	2.790	0.346	0.7338
	Isolated housing condition x pre-SxAT forced mounting duration	-1.436	2.852	-0.503	0.6216
Threats duration	Isolated housing condition	8.606	20.829	0.413	0.6850
	Pre-SxAT threats duration	-3.689	3.750	-0.984	0.3400
	Isolated housing condition x pre-SxAT threats duration	3.362	3.775	0.891	0.3860
Keep down duration	Isolated housing condition	52.137	29.267	1.781	0.0938
	Pre-SxAT keep down duration	-0.144	3.659	-0.039	0.9691
	Isolated housing condition x pre-SxAT keep down duration	0.300	3.673	0.082	0.9359
Offensive grooming duration	Isolated housing condition	88.970	26.699	3.332	0.0042
	Pre-SxAT offensive grooming duration	-0.852	0.942	-0.905	0.3788
	Isolated housing condition x pre-SxAT offensive grooming duration	0.099	1.078	0.092	0.9277
Attacks duration	Isolated housing condition	66.082	53.442	1.237	0.2340
	Pre-SxAT attacks duration	1.420	1.756	0.808	0.4310
	Isolated housing condition x pre-SxAT attacks duration	-1.396	1.776	-0.786	0.4430
Latency to attack	Isolated housing condition	-248.079	75.477	-3.287	0.0047**
	Pre-SxAT latency to attack	-0.257	0.224	-1.144	0.2694
	Isolated housing condition x pre-SxAT latency to attack	0.185	0.470	0.394	0.6986

Regression analyses assessed the influence of housing in isolation (ISM vs. GHSM, *n* = 10/group) on aggressive behaviours following the SxAT. Housing condition (isolation or group-housed), the specific behaviour measured in the resident intruder paradigm conducted before the SxAT (pre-SxAT), and their interaction were assessed as predictors for each parameter. The estimate, standard error, t-value, and p-value are provided, along with symbols indicating statistical significance levels are provided for each coefficient. *p < 0.05, **p < 0.01

### Housing in isolation increased the duration of aggressive behaviour during the four-day SxAT paradigm

**Fig. 4:**
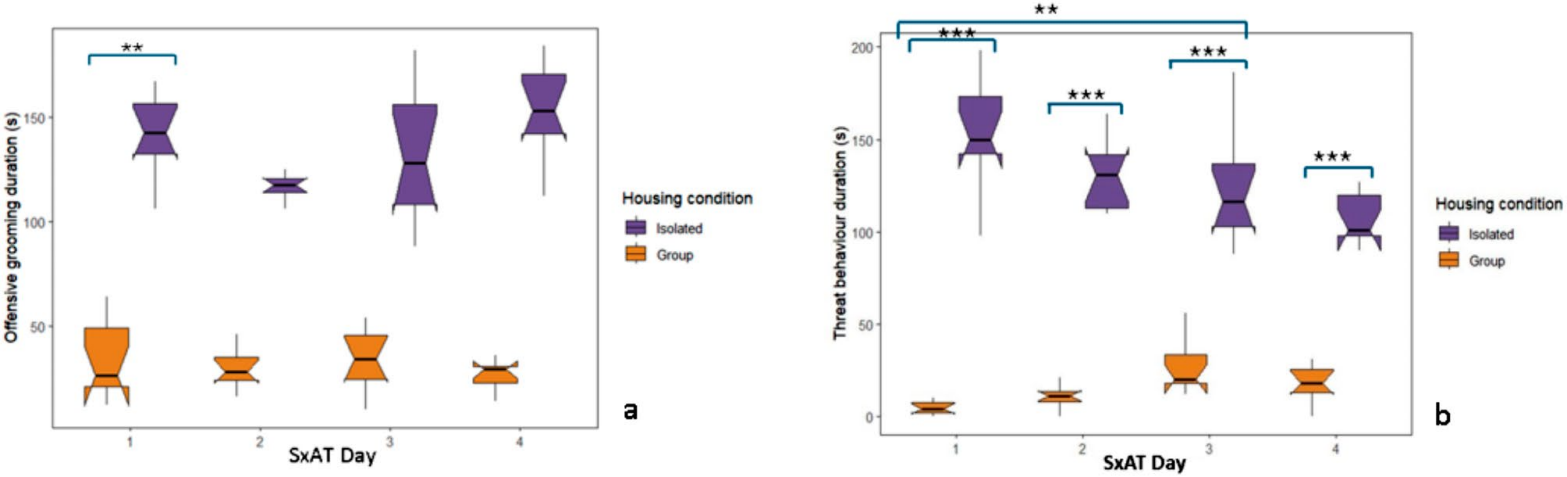
Isolation housing increased the time spent engaged in aggressive behaviour across the four-day SxAT paradigm. Isolated rats (ISM, *n* = 10) exhibited significantly longer (**a**) offensive grooming and (**b**) threat behaviour duration compared to group-housed rats (GHSM, *n* = 10). For threat duration, models indicated a significant day 3 effect and significant interactions between housing condition and day 3. Box plots represent the median and interquartile range, with whiskers for values 1.5x the interquartile range and notches for the 95% confidence interval. **p < 0.01, *** *p* < 0.001

In the SxAT paradigm, rats housed in isolation (ISM) spent more time engaged in offensive grooming than those housed in groups (GHSM) (beta estimate = 107.500, standard error = 7.839, t = 13.713, *p* < 0.001) (Fig. 4a). The analysis revealed no significant variations or interactions between the day of the test and housing condition. Rats housed in isolation also displayed significantly prolonged durations of threat behaviour compared to those housed in groups (beta estimate = 149.900, standard error = 8.346, t = 17.960, *p* < 0.001). Analyses indicated a significant effect of day 3 and day 3 x isolation group on behaviour, with post-hoc analysis indicating that this was driven by an increase in threat behaviour duration on day 3 compared to day 1 in group-housed animals (Fig. 4b).

### Housing in isolation increased anxiety-like behaviour in the EPM

**Fig. 5:**
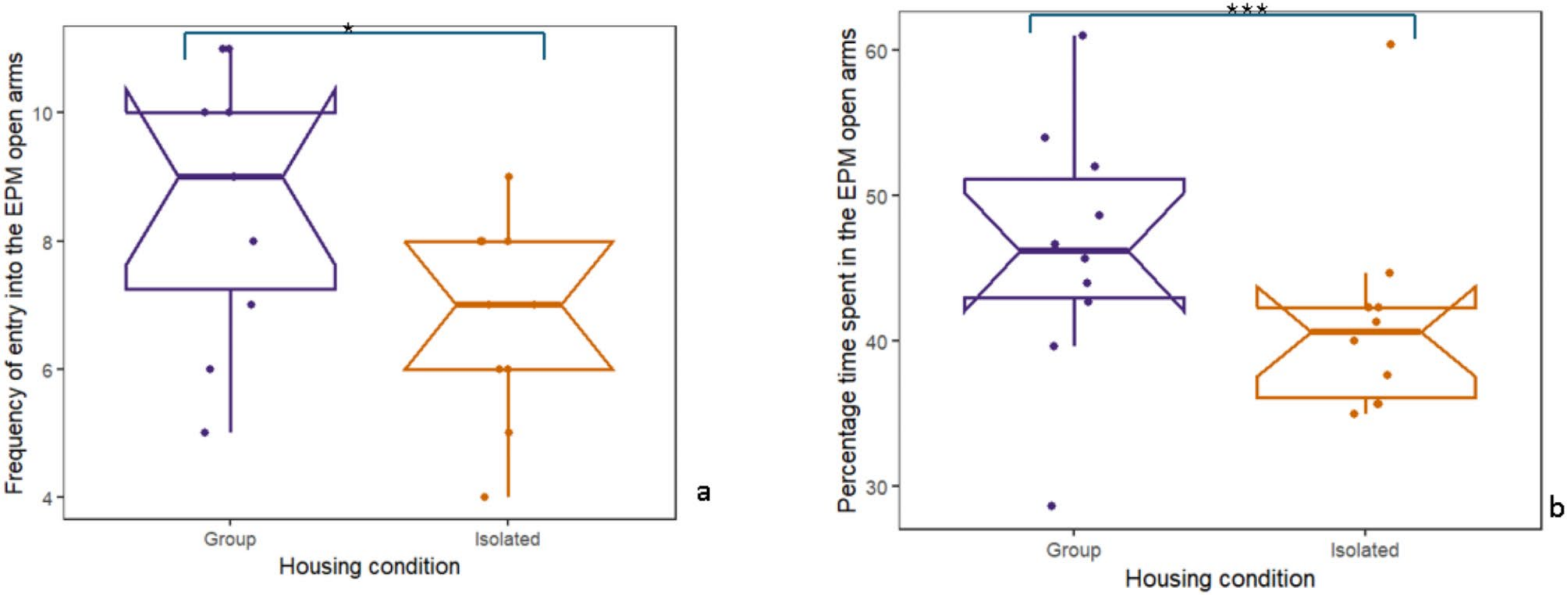
Isolation housing increased the time spent in anxiety-like behaviour in the EPM. Rats housed in isolation (ISM, *n* = 10) (**a**) entered the open arms less frequently than rats housed in groups (GHSM, *n* = 10) and (**b**) spent less time in the open arms. Raw data points are plotted, with box plots representing the median, interquartile range, whiskers for values 1.5x the interquartile range, and notches for the 95% confidence interval. *p < 0.05, **p < 0.0001

Significant differences were observed between housing condition groups in anxiety-like behaviour in rats assessed 48 h (on PND 76) after being subjected to SxAT (Table 3). Isolated (ISM) rats exhibited a significant decrease in OFT rearing frequency (t = -2.865, *p* = 0.0103) and a significant increase in OFT rearing time (t = -3.197, *p* = 0.0063) compared to group-housed (GHSM) rats. In the EPM, group-housed rats demonstrated higher EPM open arm frequency (t = 2.205, *p* = 0.0418) (Fig. 5a) and significantly more time in the open arm (W = 341, *p* = 0.0001) (Fig. 5b).

**Table 3:** Impact of isolation housing on anxiety-like behaviour in rats exposed to the SxAT

Behavioural parameter	Test statistic	Group-housed mean (SD)	Housed in isolation mean (SD)	dF	*P*-value
OFT rearing frequency ^#^	t = -2.865	9.8 ± 8.2	13.5 ± 14.3	17.934	0.0103*
OFT outer zone time ^#^	t = 0.620	172.4 ± 10.2	166.4 ± 14.8	14.124	0.5453
OFT centre zone time ^#^	t = -0.174	117.5 ± 13.0	119.3 ± 11.8	10.83	0.8650
OFT rearing time ^#^	t = -3.197	26.9 ± 8.2	43.6 ± 14.3	14.354	0.0063**
EPM open arm frequency ^#^	t = 2.205	7.9 ± 2.1	5.0 ± 1.9	16.691	0.0418*
EPM open arm time (%) ^@^	W = 341	47.7 (43.7–53.0)	35.3 (12.9–41.6)	-	0.0001***

Table 3 summarises the analysis of anxiety-like behaviours by GHSM (*n* = 10) and ISM (*n* = 10) rats in the OFT and EPM 48 h (PND 76) after the SxAT. The table includes test statistics, mean and standard deviation (SD) values, degrees of freedom (dF) where applicable, and the significance level (p-value) of the observed differences. ^#^Parametrically distributed data are reported as mean and standard deviation and were compared using the Welch two sample t-test. ^@^Non-parametric data are reported as the median and 25th and 75th percentile values and were compared using the Wilcoxon rank sum test. Significant findings are observed in OFT rearing frequency and rearing time, as well as the percentage time spent in and frequency of entry into the EPM open arms. *p < 0.05, ** *p* < 0.01, *p* < 0.001.

### Relationships between neurochemical measures and behaviours

We investigated the correlations between six neurochemical markers (serotonin, AVP, CRH, corticosterone, testosterone, and oxytocin) and anxiety-like behaviour in the OFP and EPM, as well as aggressive behaviours in the resident intruder paradigm conducted pre and post the SxAT paradigm.

### Neurochemical marker levels are associated with aggressive behaviour in the resident intruder paradigm

Analyses indicated significant correlations between various neurochemical markers and aggressive behaviour duration in the pre-SxAT resident intruder paradigm, among ISM and GHSM groups, (Table 4). Serotonin (Spearman’s rho = -0.464, *p* = 0.0128) and CRH (Spearman’s rho = -0.419, *p* = 0.0263) exhibited moderate while oxytocin (Spearman’s rho = -0.721, *p* = 0.0000) showed strong negative correlations with aggressive behaviour duration. In contrast, corticosterone (Spearman’s rho = 0.549, *p* = 0.0025) and AVP (Spearman’s rho = 0.791, *p* = 0.0000) showed moderate and strong positive correlations, respectively. In the post SxAT correlation analysis, higher levels of serotonin were significantly associated with reduced aggression duration (Spearman’s rho = -0.603, *p* = 0.0225). Higher oxytocin levels were strongly associated with shorter durations of aggressive behaviour (Spearman’s rho = -0.684, *p* = 0.0070). In contrast, AVP exhibited a strong positive correlation with aggressive behaviour duration (Spearman’s rho = 0.698, *p* = 0.0055) (Table 4). Raw group data are included in supplementary Table 2.

**Table 4:** Correlations between serum levels of neurochemical markers and aggressive behaviour duration before and after the SxAT paradigm

Neurochemical marker	Pre-SxAT		Post-SxAT	
	Correlation coefficient (Spearman’s rho)	*P*-value	Correlation coefficient (Spearman’s rho)	*P*-value
Testosterone	0.105	0.5964	0.189	0.5171
Serotonin	-0.464	0.0128*	-0.602	0.0225*
Corticosterone	0.549	0.0025**	0.396	0.1610
Oxytocin	-0.721	0.0001***	-0.684	0.0070**
AVP	0.791	0.0001***	0.698	0.0055**
CRH	-0.419	0.0263*	0.068	0.8168

AVP = arginine vasopressin, CRH = corticotropin-releasing hormone, SxAT = sexual aggression test. *p < 0.05, **p < 0.01, *** *p* < 0.001

### Neurochemical marker levels are associated with aggressive behaviour in the SxAT

The correlations between serum levels of various neurochemical markers and the percentage time spent in aggressive behaviour during the SxAT revealed significant relationships (Table 5). AVP (Spearman’s rho = 0.674, *p* = 0.0082) and corticosterone (Spearman’s rho = 0.537, *p* = 0.0478) were moderately positively correlated with the duration of aggressive behaviour. Testosterone also showed a moderate positive correlation with aggressive behaviour but only at a nominally significant level (Spearman’s rho = 0.517, *p* = 0.0583). On the other hand, serotonin (Spearman’s rho = -0.552, *p* = 0.0406) and oxytocin (Spearman’s rho = -0.810, *p* = 0.0004) were moderately and very strong negatively correlated with aggressive behaviour, respectively.

**Table 5:** Correlations between serum levels of neurochemical markers and percentage time spent in aggressive behaviour during the SxAT paradigm

Neurochemical marker	Correlation coefficient (Spearman’s rho)	*P*-value
Testosterone	0.517	0.0583
Serotonin	0.552	0.0406*
Corticosterone	0.537	0.0478*
Oxytocin	-0.810	0.0004 ***
AVP	0.674	0.0082**
CRH	-0.207	0.4781

AVP = arginine vasopressin, CRH = corticotropin releasing hormone. *p < 0.05, **p < 0.01, *** *p* < 0.001

### Neurochemical marker levels are associated with anxiety-like behaviours

The correlation analysis between neurochemical markers and anxiety-like behaviours in the OFT and EPM revealed significant relationships that provide insights into the neurobiological basis of these behaviours (Table 6). Higher serum levels of oxytocin (Spearman’s rho = -0.571, *p* = 0.0015) and CRF (Spearman’s rho = -0.661, *p* = 0.0001) were moderately and strongly associated with decreased time spent in the OFT outer zone, respectively. On the other hand, higher levels of AVP were moderately correlated with increased time spent in the outer zone of the OFT (Pearson’s *r* = 0.524, *p* = 0.0042). In the EPM (Table 7), higher serum levels of serotonin were moderately associated with a longer time spent in the open arm (rho = 0.421, *p* = 0.0256). Higher levels of serum corticosterone (Spearman’s rho = -0.559, *p* = 0.0020) and AVP (Spearman’s rho = -0.617, *p* = 0.0005) were moderately correlated with less time spent in the open arm. On the other hand, higher levels of oxytocin were significantly and strongly correlated with more time spent in the open arms (Spearman’s rho = 0.698, *p* = 0.0000).

**Table 6:** Correlation between serum levels of neurochemical markers and anxiety-like behaviour in the OFT

Neurochemical marker	OFT outer zone time		OFT centre zone time	
	Correlation coefficient	*P*-value	Correlation coefficient	*P*-value
Testosterone ^@^	-0.157	0.4237	0.126	0.5230
Serotonin ^#^	0.003	0.9871	0.093	0.6393
Corticosterone ^@^	0.369	0.0535	-0.409	0.0305*
Oxytocin ^@^	-0.571	0.0015**	0.630	0.0003***
AVP ^#^	0.524	0.0042**	-0.580	0.0012**
CRH ^@^	-0.661	0.0001***	0.631	0.0003***

^#^Parametric data report Pearson’s r and ^@^ non-parametric data report Spearman’s rho. AVP = arginine vasopressin, CRH = corticotropin-releasing hormone. *p < 0.05, **p < 0.01, ***p < 0.001

**Table 7:** Correlation between serum levels of neurochemical markers and anxiety-like behaviour in the EPM

Neurochemical marker	Behavioural parameter	Correlation coefficient (Spearman’s rho)	*P*-value
Testosterone	EPM % open arm time	0.309	0.1096
Serotonin	EPM % open arm time	0.421	0.0256*
Corticosterone	EPM % open arm time	-0.559	0.0020**
Oxytocin	EPM % open arm time	0.698	0.0000***
AVP	EPM % open arm time	-0.617	0.0005***
CRH	EPM % open arm time	0.408	0.0313*

AVP = arginine vasopressin, CRH = corticotropin-releasing hormone. *p < 0.05, **p < 0.01, ***p < 0.001

### Analysis of the relationship between isolation housing, resident-intruder aggressive behaviour and gene expression in specific brain regions

We performed linear regression analyses to explore how housing condition and the duration of aggressive behaviour prior to sexual aggression influence the expression of key neurochemical markers, including *CRHR1*, *HTR1A*, *AVP*, *OXTR*, *AVPR1A*, and *AR*, across different brain regions: the PFC, HYPO, HIPP, and AMYG. These analyses aimed to elucidate the complex interplay between environmental and behavioural factors and their neurobiological underpinnings in the context of aggression and stress response.

**Fig. 6:**
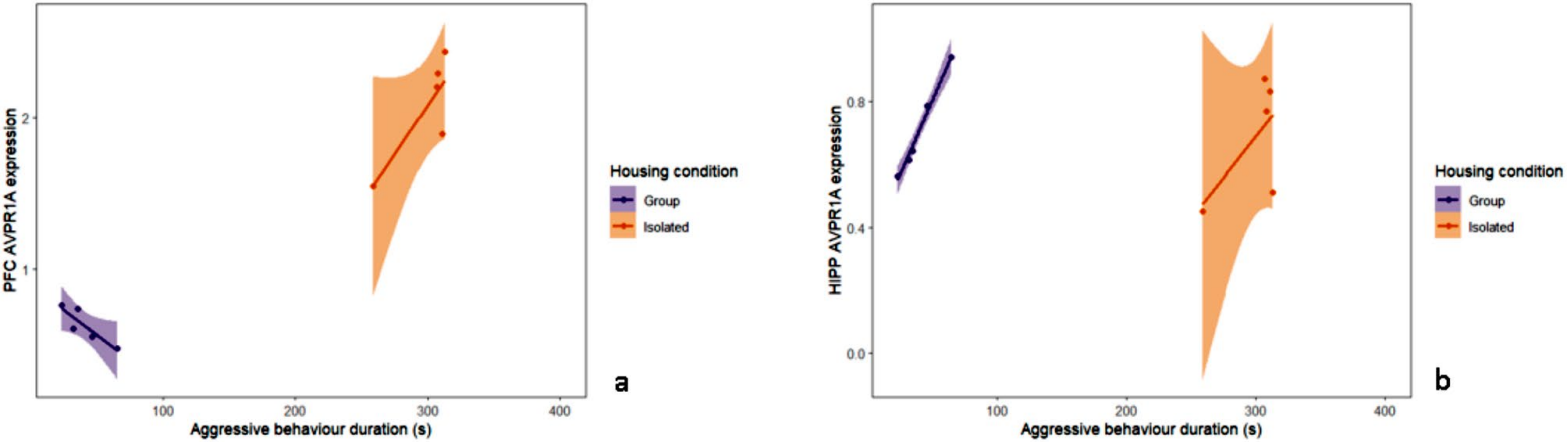
*AVPR1A* expression is influenced by isolation housing and the duration of aggressive behaviour displayed in the pre-SxAT resident intruder test. (**a**) *AVPR1A* expression in the PFC is nominally associated with isolation housing and significantly associated with the interaction between isolation housing and aggressive behaviour duration. (**b**) In the hippocampus, longer pre-SxAT aggression duration was positively associated with *AVPR1A* expression. Graphs display the regression line and 95% confidence interval. *N* = 5/group. HIPP = hippocampus, PFC = prefrontal cortex

The linear regression analyses assessing the impact of isolation vs. group housing condition and the duration of aggressive behaviour on the expression of various neurochemical markers revealed notable findings, particularly with *AVPR1A* expression in the PFC and HIPP (Table 8). The model indicated a nominal effect of housing condition (beta estimate = -2.683, standard error = 1.124, t = -2.388, *p* = 0.0542) and a significant housing condition x pre-SxAT aggressive behaviour interaction effect (beta estimate = 0.019, standard error = 0.006, t = 3.058, *p* = 0.0220), where *AVPR1A* expression was positively associated with aggressive behaviour in the rats exposed to social isolation but inversely correlated in group-housed rats (Fig. 6a). *AVPR1A* expression in the hippocampus was positively associated with pre-SxAT aggressive behaviour (beta estimate = 0.010, standard error = 0.004, t = -2.448, *p* = 0.0499) (Fig. 6b).

**Table 8:** Impact of housing condition and aggressive behaviour duration on brain neurochemical marker expression

Brain region	Model coefficients	Estimate	Standard error	t value	Pr(>|t|)
*CRHR1*					
PFC	Isolated housing condition	1.504	1.314	-1.145	0.2958
	Pre-SxAT aggressive behaviour duration	-0.002	0.006	-0.248	0.8126
	Isolated housing condition x pre-SxAT aggressive behaviour duration	-1.436	2.852	-0.503	0.6216
HYPO	Isolated housing condition	2.048	3.941	0.520	0.6220
	Pre-SxAT aggressive behaviour duration	-0.011	0.018	-0.590	0.5770
	Isolated housing condition x pre-SxAT aggressive behaviour duration	0.006	0.022	0.251	0.8100
HIPP	Isolated housing condition	1.350	1.457	-0.926	0.3900
	PreSxAT aggressive behaviour duration	-0.002	0.007	-0.294	0.7785
	Isolated housing condition x Pre-SxAT aggressive behaviour duration	0.006	0.008	0.741	0.4865
AMYG	Isolated housing condition	1.469	1.332	1.103	0.3122
	Pre-SxAT aggressive behaviour duration	0.001	0.006	0.231	0.8250
	Isolated housing condition x pre-SxAT aggressive behaviour duration	-0.006	0.008	-0.800	0.4544
*HTR1A*					
PFC	Isolated housing condition	-5.172	3.937	-1.314	0.2369
	Pre-SxAT aggressive behaviour duration	-0.039	0.018	-2.147	0.0754
	Isolated housing condition x pre-SxAT aggressive behaviour duration	0.051	0.022	2.300	0.0611
HYPO	Isolated housing condition	0.589	1.687	0.349	0.7388
	Pre-SxAT aggressive behaviour duration	0.003	0.008	0.419	0.6900
	Isolated housing condition x pre-SxAT aggressive behaviour duration	-0.004	0.010	-0.424	0.6862
HIPP	Isolated housing condition	3.155	4.045	0.780	0.4650
	Pre-SxAT aggressive behaviour duration	-0.005	0.019	-0.258	0.8050
	Isolated housing condition x pre-SxAT aggressive behaviour duration	-0.006	0.023	-0.261	0.8030
AMYG	Isolated housing condition	-2.094	4.276	-0.490	0.6420
	Pre-SxAT aggressive behaviour duration	-0.003	0.020	-0.156	0.8810
	Isolated housing condition x pre-SxAT aggressive behaviour duration	0.010	0.024	0.423	0.6870
*OXTR*					
PFC	Isolated housing condition	2.441	4.218	0.579	0.5839
	Pre-SxAT aggressive behaviour duration	-0.002	0.020	-0.097	0.9259
	Isolated housing condition x pre-SxAT aggressive behaviour duration	-0.009	0.024	-0.361	0.7305
HYPO	Isolated housing condition	-1.345	2.115	-0.636	0.5483
	Pre-SxAT aggressive behaviour duration	-0.003	0.010	-0.332	0.7512
	Isolated housing condition x pre-SxAT aggressive behaviour duration	0.006	0.012	0.460	0.6615
HIPP	Isolated housing condition	-2.881	5.831	-0.494	0.6390
	Pre-SxAT aggressive behaviour duration	0.032	0.027	1.173	0.2850
	Isolated housing condition x pre-SxAT aggressive behaviour duration	-0.018	0.033	-0.548	0.6040
AMYG	Isolated housing condition	0.292	2.328	0.125	0.9042
	Pre-SxAT aggressive behaviour duration	-0.011	0.011	-0.998	0.3570
	Isolated housing condition x pre-SxAT aggressive behaviour duration	0.008	0.013	0.639	0.5465
*AR*					
PFC	Isolated housing condition	1.019	0.592	1.721	0.1361
	Pre-SxAT aggressive behaviour duration	-0.004	0.003	-1.410	0.2081
	Isolated housing condition x pre-SxAT aggressive behaviour duration	0.000	0.003	0.071	0.9457
HYPO	Isolated housing condition	0.115	0.733	0.157	0.8808
	Pre-SxAT aggressive behaviour duration	0.005	0.003	1.607	0.1591
	Isolated housing condition x pre-SxAT aggressive behaviour duration	-0.006	0.004	-1.337	0.2297
HIPP	Isolated housing condition	-1.650	2.064	-0.800	0.4543
	Pre-SxAT aggressive behaviour duration	0.012	0.010	1.234	0.2633
	Isolated housing condition x pre-SxAT aggressive behaviour duration	-0.006	0.012	-0.475	0.6517
AMYG	Isolated housing condition	-0.366	1.593	-0.230	0.8261
	Pre-SxAT aggressive behaviour duration	-0.006	0.007	-0.832	0.4373
	Isolated housing condition x pre-SxAT aggressive behaviour duration	0.008	0.009	0.837	0.4348
*AVPR1A*					
PFC	Isolated housing condition	-2.683	1.124	-2.388	0.0542
	Pre-SxAT aggressive behaviour duration	-0.007	0.005	-1.263	0.2535
	Isolated housing condition x pre-SxAT aggressive behaviour duration	0.019	0.006	3.058	0.0223*
HYPO	Isolated housing condition	3.225	2.414	1.336	0.2300
	Pre-SxAT aggressive behaviour duration	0.004	0.011	0.364	0.7280
	Isolated housing condition x pre-SxAT aggressive behaviour duration	-0.012	0.014	-0.840	0.4330
HIPP	Isolated housing condition	-1.220	0.836	-1.459	0.1948
	Pre-SxAT aggressive behaviour duration	0.010	0.004	2.448	0.0499*
	Isolated housing condition x pre-SxAT aggressive behaviour duration	-0.004	0.005	0.888	0.4087
AMYG	Isolated housing condition	-1.488	2.066	-0.720	0.4990
	Pre-SxAT aggressive behaviour duration	-0.002	0.010	-0.186	0.8590
	Isolated housing condition x pre-SxAT aggressive behaviour duration	0.008	0.012	0.713	0.5030

Regression analyses assessed the influence of housing in isolation (ISM vs. GHSM, *n* = 5/group) and post-SxAT aggressive behaviour duration affect gene expression (CRHR1, HTR1A, AVP, OXTR, AR and AVPR1A) in four brain regions (PFC, HYPO, HIPP, AMYG) of adult male rats. The estimate, standard error, t-value, and p-value are provided, along with symbols indicating statistical significance levels are provided for each coefficient. PFC = prefrontal cortex, HYPO = hypothalamus, HIPP = hippocampus, AMYG = amygdala. *p < 0.05

### Analysis of the relationship between isolation housing, sexual aggression and gene expression in specific brain regions

**Fig. 7:**
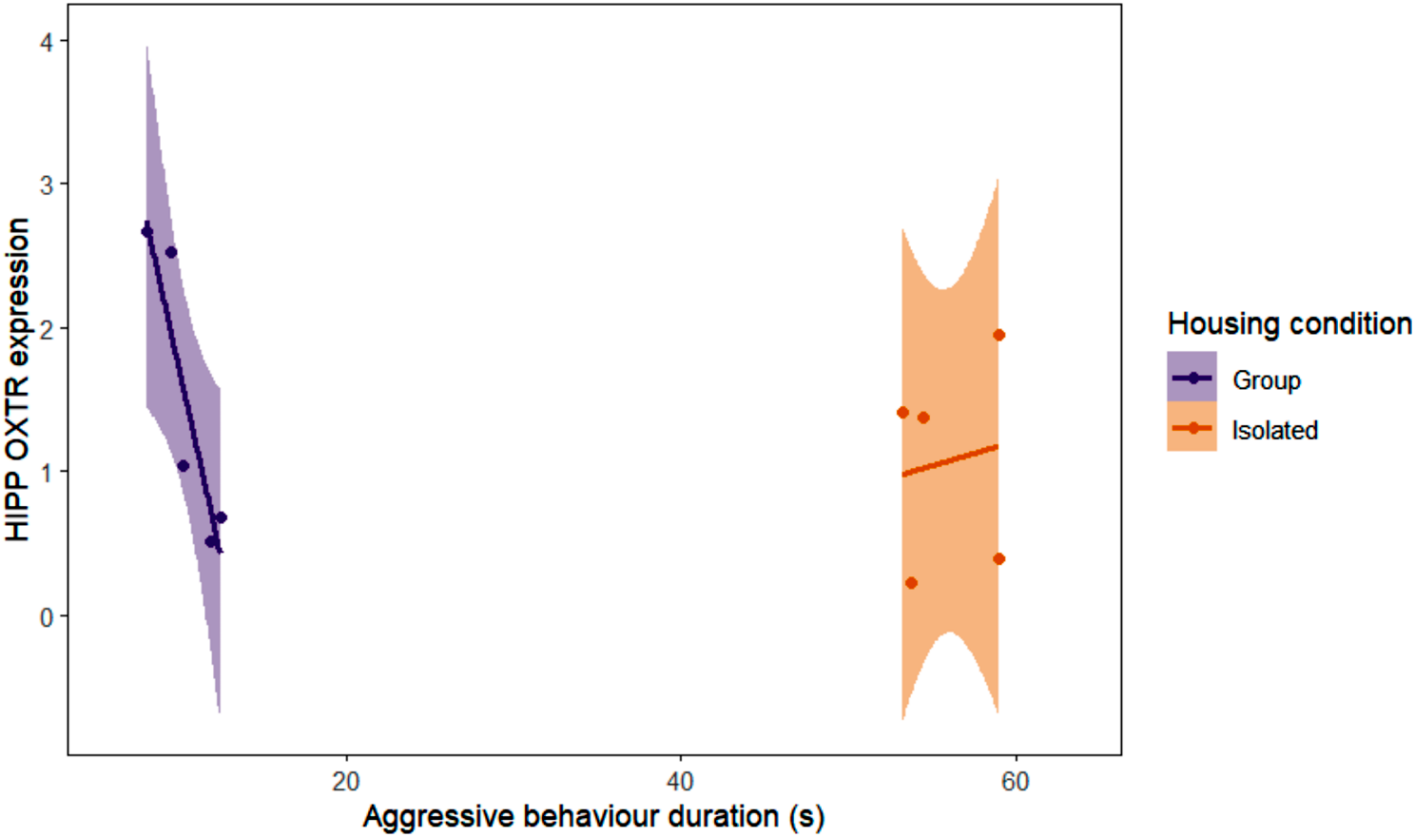
Impact of housing condition and sexual aggression behaviour duration on OXTR expression in the hippocampus. In GHSM rats, increased aggression exposure was associated with reduced *OXTR* expression compared to ISM rats (*n* = 5/group). The interaction of housing and sexual aggression duration significantly modulated OXTR levels. Graphs display the regression line and 95% confidence interval. HIPP = hippocampus

We also analysed the relationship between housing condition, the duration of sexual aggression behaviour, and gene expression levels. The expression of *OXTR* in the hippocampus was significantly associated with the interaction between housing condition and the percentage of time spent engaged in sexually aggressive behaviour (Fig. 7). In GHSM, increased aggression exposure was associated with reduced expression (beta estimate =-0.522 standard error = 0.193, t = -2.708, *p* = 0.0352). However, the significant interaction (beta estimate = 0.556, standard error = 0.227, t = 2.448, *p* = 0.0499) effect indicated that the negative impact of aggression on OXTR expression was offset when combined with the isolation housing condition.

## Discussion

The study aimed to understand the complex dynamics underlying sexual aggression using the SxAT paradigm. The study focused on experimentally inducing aggressive behaviour in male rats by disrupting the HPA-axis through social isolation [50–52]. The resident intruder test was employed to examine the manifestation of these behaviours. Subsequently, we assessed whether the induced aggressive behaviour translated into sexual aggression towards non-receptive females. The SxAT was used to understand the dynamics and determinants of aggressive behaviours, specifically in the context of mating or sexual encounters. It allowed us to observe, quantify, and analyse aggressive behaviours displayed during unwanted sexual interactions. To uncover the neurochemical underpinnings of sexual aggression, the study analysed changes in multiple neurotransmitters and hormones, including CRH, oxytocin, AVP, serotonin, testosterone, and corticosterone. Concurrently, expression levels of *OXTR*, *CHRH1*, *5-HTR1A*, *AVP1A*, and *AR*, were detected and quantified in specific brain regions, namely the hypothalamus, prefrontal cortex, hippocampus, and amygdala.

We explored the effects of housing conditions on aggressive behaviour in male Wistar rats before exposure to the SxAT. Our findings demonstrated a clear and significant increase in aggressive behaviours, such as forced mounting, threats, keep down, offensive grooming, and attack durations, among isolated rats compared to their group-housed counterparts. These results are consistent with previous research indicating that social isolation can exacerbate aggressive tendencies in rodents [53, 54]. These findings also align with Wongwitdecha & Marsden (1996), who observed that social isolation not only intensifies aggressive behaviour but also modulates the effects of diazepam [55]. The activation of the HPA axis in response to social isolation, leading to the release of stress hormones and subsequent behavioural adaptations, is suggested to contribute to the neuroendocrine basis of aggressive behaviour. The chronic activation of the HPA axis and resulting hormonal imbalances are implicated in dysregulating emotional states and increasing aggressive tendencies [56, 57]. Our regression analysis of post-SxAT behaviours highlighted the significant main effects of housing conditions on “offensive grooming” and “forced mounting” durations, and shorter latency to the first attack, indicating that isolation exacerbates aggressive responses even post-sexual aggression. These findings align with previous research emphasising the long-lasting impact of social isolation on aggression modulation [58]. These results also support the notion that social isolation not only impacts initial aggressive behaviours but also influences subsequent interactions, potentially through alterations in stress and aggression-related neurobiological pathways [59–61].

Our resident intruder test findings suggest that social isolation plays a pivotal role in shaping aggressive behaviour with potential implications for understanding the neurobiological mechanisms driving these behaviours. Changes in stress-responsive systems and disruptions in neurotransmitter pathways could underlie the observed behavioural alterations, highlighting the profound impact of social experiences on aggression regulation. Our analysis revealed that serum serotonin, oxytocin and CRH exhibited negative correlations with the duration of aggressive behaviour in the pre-SxAT resident intruder test, with serotonin and oxytocin continuing to show this relationship post-SxAT, aligning with previous research highlighting their inhibitory effects on aggression [62, 63]. Conversely, serum corticosterone and AVP showed positive correlations with aggression duration, suggesting that these markers may facilitate or exacerbate aggressive tendencies. The role of AVP in promoting aggression has been well-documented, with AVP acting as a neuromodulator in aggression-related brain areas [64, 65]. For instance, Ferris et al. (1984) demonstrated that vasopressin injections into the hypothalamus could trigger aggressive behaviours in golden hamsters, marking one of the first studies to establish this relationship [66]. Additionally, research by Albers & Ferris (1986) provided further evidence for the involvement of vasopressin, along with substance P, in the regulation of aggression in golden hamsters, highlighting the neurochemical pathways implicated in aggressive behaviours [67]. Corticosterone’s positive correlation with aggression duration may reflect its broader role in stress and arousal, which can underpin aggressive behaviours [68, 69].

Our findings are in line with existing research indicating the modulatory role of serotonin in aggression [70]. Aggression levels has been correlated with *5-HT1A* receptor availability in specific brain regions, including the prefrontal and anterior cingulate cortices, with higher aggression associated with increased receptor availability in these areas. Analysis of aggressive behaviour in the post-SxAT resident intruder test further highlights the inverse relationship between serotonin and aggressive behaviour duration, aligning with the serotonin deficiency hypothesis of aggression [71]. Oxytocin’s negative correlation with post-SxAT aggressive behaviour supports its role in reducing aggression, echoing findings that link oxytocin to prosocial behaviours [72]. The strongest positive correlation was observed between AVP and aggressive behaviour, with this positive correlation continuing in post-SxAT analyses, suggesting a consistent influence on aggression. This underscores the potential involvement of AVP in regulating aggression [73], with this effect potentially occurring via the vasopressinergic system’s modulation of social behaviours [74].

In addition to serum markers, we explored how housing conditions and pre-SxAT aggressive behaviour affected key neurochemical markers’ expression in specific brain regions. *AVPR1A* expression in the prefrontal cortex and hippocampus was significantly influenced by the interaction between housing conditions and aggressive behaviour. In the prefrontal cortex, the model highlighted a notable interaction effect between housing conditions and aggressive behaviour on *AVPR1A* expression, indicating that social isolation positively correlates with AVPR1A expression, linked to aggressive behaviour. This is consistent with findings that suggest that social and aggressive behaviours can lead to alteration in *AVPR1A* levels [75, 76]. AVPR1A plays a role in modulating social recognition and memory processes [77]. The prefrontal cortex is involved in higher cognitive functions, including decision-making and social behaviour regulation [78]. AVPR1A receptors in the prefrontal cortex influence these functions by affecting the processing of social information and the formation of social memories [79]. Disruption of these processes could lead to alterations in social behaviour, including increased aggression. AVPR1A has also been shown to interact with other neurotransmitter systems, such as serotonin and dopamine, which are known to play roles in regulating aggression [80]. AVPR1A activation in the PFC may enhance dopamine release, which has been implicated in promoting aggressive behaviour [81, 82]. The AVPR1A receptor may also interact with oxytocin receptors in the brain, as both play crucial roles in regulating social behaviours and stress responses [83, 84]. This interaction may influence the expression of aggressive behaviour, with social isolation potentially altering this interaction and leading to increased aggression in some individuals. A previous study showed a significant correlation between *AVPR1A* expression in the hippocampus and aggressive behaviour in mice [75]. The study showed that increased *AVPR1A* expression in the hippocampus is associated with heightened aggressive behaviour. The hippocampus plays a critical role in memory formation and stress responses, and AVPR1A influences these processes by modulating the release of vasopressin, a neuropeptide implicated in social behaviour regulation [85]. Enhanced *AVPR1A* expression could lead to increased vasopressin signalling, which has been linked to aggressive responses, particularly in stressful or social dominance scenarios. The analysis of the impact of sexual aggression on *OXTR* expression in the hippocampus revealed that increased aggression exposure is linked to reduced *OXTR* expression in group-housed rats. This effect was offset in isolated rats, suggesting a protective or compensatory mechanism elicited by oxytocin in the context of social isolation. This is in line with research indicating oxytocin’s role in promoting social bonding and mitigating stress responses [86–88], emphasising its potential as a modulator of aggression and social interaction in adverse environments.

With respect to sexual aggression, we also found effects of housing condition on behaviour. Rats housed in isolation also spent more time engaged in offensive grooming and threat behaviour in the SxAT compared to group-housed rats, supporting the value of this animal model of sexual aggression. We found significant correlations between serum levels of certain neurochemicals and the percentage of time spent in aggressive behaviour during the SxAT, supporting the role of these neurochemicals in modulating sexual aggression. Consistent with the resident intruder aggression findings, AVP and corticosterone both showed significant positive correlations with aggression during a sexually aggressive encounter. Research has consistently shown that AVP plays a significant role in modulating aggressive behaviour, particularly in sexual and territorial contexts. In animal studies, AVP has been found to increase aggression in sexually naive male hamsters and male squirrel monkeys, especially when normal olfactory cues are disrupted, suggesting its involvement in sexual competition and mating behaviour [14, 89]. Additionally, in monogamous rodents, AVP in the ventral tegmental area has been linked to heightened aggression related to mating and territorial defence [90]. Human studies further support this, showing that higher cerebrospinal fluid levels of AVP correlate with increased aggression in individuals with personality disorders, highlighting AVP’s broader role in aggressive behaviours [91]. Elevated corticosterone levels have been linked to increased aggression in male rodents, especially following social defeat and under stress, suggesting a key role for this hormone in sexual aggression [92]. Corticosterone influences brain regions such as the amygdala and hypothalamus, which are critical for aggressive behaviours, as discussed in comprehensive reviews of the HPA axis’s involvement in aggression [93, 94]. Moreover, corticosterone’s role in enhancing sexual aggression, particularly under stress or competitive conditions, has been further supported by studies on sexual motivation and behaviour [95, 96]. Combined, these results indicate that both AVP and corticosterone are implicated in aggressive behaviour more broadly, and, of relevance to this study, in sexually aggressive behaviour.

The significant negative correlations observed for serotonin and oxytocin in the SxAT suggest that these neurochemicals may serve as inhibitory modulators of sexual aggression and are in accordance with the inverse correlations with aggressive behaviour observed in the resident intruder test. Previous findings have shown that serotonin plays a critical inhibitory role in aggressive behaviour, including sexual aggression. Studies have demonstrated that serotonin, particularly through its action in brain areas like the hypothalamus and prefrontal cortex, generally suppresses aggression [74]. In animal models, elevated serotonin levels are linked to reduced aggression, while lower serotonin levels, often influenced by genetic factors such as the MAO-A gene, are associated with increased aggression [97]. The modulation of aggression through specific serotonin receptors (5-HT1A and 5-HT1B) further supports its inhibitory function in both animals and humans [98]. Additionally, lower levels of serotonin metabolites in cerebrospinal fluid have been correlated with higher rates of aggressive behaviours in nonhuman primates, reinforcing serotonin’s role as a key regulator of aggression [99]. Studies in male rats and prairie voles have demonstrated that oxytocin reduces aggressive behaviour, particularly following sexual experiences, by enhancing anti-aggressive and social bonding behaviours [100]. In humans, oxytocin has been found to reduce aggressive responses, especially in social or sexual conflict situations [101]. However, in individuals with high trait aggression, oxytocin can paradoxically increase tendencies toward intimate partner violence, suggesting a complex and context-dependent role of oxytocin in modulating aggression [20].

In our study on anxiety-like behaviour in rats post-SxAT, we observed notable differences influenced by their housing conditions. Isolated rats showed less frequent rearing in the OFT, suggesting heightened anxiety, as rearing is typically associated with a sense of security [102]. This behaviour aligns with previous findings that prolonged isolation can increase anxiety in rodents [103, 104]. Additionally, these isolated rats spent more time rearing, potentially indicating increased anxiety due to the absence of social buffering available to group-housed rats. In the EPM, group-housed rats showed significantly higher frequency of entries to the open arms and spent more time in the open arms compared to their isolated counterparts. These findings align with established literature where increased time and frequency in the open arms of the EPM are indicative of reduced anxiety levels [105]. These results highlight how isolation can exacerbate anxiety-like behaviours in rats, evident from the increased rearing time and decreased open arm time in the OFT and EPM, respectively. On the other hand, group housing seemed to mitigate anxiety, as indicated by the increased open arm frequency in the EPM. These findings are consistent with broader research suggesting that social isolation can significantly impact mental health, particularly by increasing anxiety and stress [106, 107].

Our findings also highlight significant correlations between serum neurochemical markers and behaviours in both the OFT and EPM. Notably, oxytocin showed a robust negative correlation with anxiety-like behaviours, underscoring its anxiolytic properties and role in promoting social and exploratory behaviours [72]. This is complemented by the negative correlations between serotonin and anxiety-like behaviours, reinforcing serotonin’s role in anxiety regulation [108, 109]. The positive correlation between AVP and anxiety-like behaviours in the OFT, coupled with its positive correlation with aggression, paints a picture of AVP as a potential modulator of stress-related behaviours, influencing both aggression and anxiety [110, 111]. Corticosterone’s positive correlation with anxiety-like behaviours in the EPM further supports its role in stress responses and anxiety [112, 113]. These findings collectively illuminate the complex neurochemical landscape underlying aggression and anxiety-like behaviours. The modulatory roles of serotonin and oxytocin, in particular, suggest potential therapeutic targets for mitigating aggression and anxiety, emphasising the importance of neurochemical balance in behavioural regulation. Furthermore, the differential effects of AVP and corticosterone on both aggression and anxiety-like behaviours highlight the nuanced and context-dependent roles these markers play in modulating behavioural responses.

### Limitations

There are several limitations in our study to that prevent a fully comprehensive understanding of our findings. First, the direct association between our rat model and human sexual aggression requires careful consideration. While animal models are invaluable for elucidating the neurobiological mechanisms underlying aggression, human sexual aggression is a multifaceted phenomenon influenced by a wide range of psychological, social, and biological factors. Therefore, our study refrains from making definitive statements about the direct applicability of our findings to human sexual aggression. Instead, we present this as a potential area for further exploration, ensuring that our study is framed within the appropriate context of animal behaviour research. Our model captures specific aspects of aggressive behaviour, and given this limitation, cannot be overgeneralised as absolutely applicable to humans.

We also acknowledge that the validity and reliability of correlations drawn from a relatively small sample size (*n* = 5), may be a concern. While this sample size is consistent with several recent studies in the field [114–117], larger sample sizes would provide more robust data and we suggest that our analyses are repeated in future studies to confirm the validity of these findings.

It would be optimal to measure the levels of OXT and AVP in the brain to draw a more direct association with behavioural changes. However, peripheral levels of these neuropeptides can reflect central neurochemical activity relevant to behavioural studies and recent studies have demonstrated correlations between peripheral measurements of these neuropeptides and behavioural outcomes [118–121].

Finally, our characterisation of the behavioural phenotype of these animals is incomplete. More insight into appetitive sexual activity, as well as a more comprehensive assessment of whether the aggression displayed is defensive, offensive or injurious is required. Such experiments could also allow for more nuanced interpretations of the neurochemical data.

## Conclusion

The study demonstrated that social isolation exacerbates sexually aggressive behaviours in male Wistar rats influenced by stress. Additionally, the study highlighted significant correlations between neurochemical markers and behaviours. Notably, oxytocin and serotonin exhibited negative correlations with aggression and anxiety-like behaviours, suggesting their potential roles in mitigating these responses. Conversely, AVP’s positive correlation with both aggression and anxiety-like behaviours underscores its modulatory role in stress-related behaviours. Social isolation was found to exacerbate aggressive behaviours and alter neurochemical marker expressions, specifically increasing *AVPR1A* expression in the prefrontal cortex and hippocampus, which correlated with heightened aggression. This aligns with prior research suggesting social isolation impacts aggression and stress responses. We observed reduced *OXTR* expression in the hippocampus with increased aggression exposure, highlighting the complex interplay between social experiences and neurobiology in aggression modulation.

## Electronic supplementary material

Below is the link to the electronic supplementary material.

Supplementary Material 1

## Data Availability

The dataset supporting the conclusions of this article is provided within the manuscript or supplementary information files.

## References

[1] World Health Organization (WHO). Guidelines for medico-legal care of victims of sexual violence. Sexual violence: Prevalence, dynamics and consequences; 2004.

[2] Graaff K, Heinecken L. Masculinities and gender-based violence in South Africa: a study of a masculinities-focused intervention programme. Dev South Afr. 2017;34(5):622–34.

[3] Du Toit L. Shifting meanings of postconflict sexual violence in South Africa. J Women Cult Soc. 2014;40(1):101–23.

[4] Liu J, Lewis G, Evans L. Understanding aggressive behavior across the Life Span. J Psychiatr Ment Health Nurs. 2013;20(2):156. 22471771 10.1111/j.1365-2850.2012.01902.x PMC3411865

[5] Korpel POJ, Varkevisser T, Hoppenbrouwers SS, Van Honk J, Geuze E. The predictive value of early-life trauma, psychopathy, and the testosterone-cortisol ratio for impulsive aggression problems in Veterans. Chronic Stress Thousand Oaks Calif. 2019;3:2470547019871901. 32440599 10.1177/2470547019871901 PMC7219916

[6] Fritz M, Soravia SM, Dudeck M, Malli L, Fakhoury M. Neurobiology of Aggression—Review of recent findings and relationship with Alcohol and Trauma. Biology. 2023;12(3):469. 36979161 10.3390/biology12030469 PMC10044835

[7] Chovatiya R, Medzhitov R, Stress. Inflammation, and Defense of Homeostasis. Mol Cell. 2014;54(2):281. 24766892 10.1016/j.molcel.2014.03.030 PMC4048989

[8] Hinds JA, Sanchez ER. The role of the hypothalamic-pituitary-adrenal (HPA) Axis in Test-Induced anxiety: assessments, physiological responses, and Molecular Details. Stress 2022. 2022;2(1):146–55.

[9] Heck AL, Handa RJ. Sex differences in the hypothalamic–pituitary–adrenal axis’ response to stress: an important role for gonadal hormones. Neuropsychopharmacology. 2019;44(1):45. 30111811 10.1038/s41386-018-0167-9 PMC6235871

[10] Leistner C, Menke A. Hypothalamic-pituitary-adrenal axis and stress. Handb Clin Neurol. 2020;175:55–64. 33008543 10.1016/B978-0-444-64123-6.00004-7

[11] Raise-Abdullahi P, Meamar M, Vafaei AA, Alizadeh M, Dadkhah M, Shafia S, et al. Hypothalamus and post-traumatic stress disorder: a review. Brain Sci. 2023;13(7):1010. 37508942 10.3390/brainsci13071010 PMC10377115

[12] Inoue Y, Burriss RP, Hasegawa T, Kiyonari T. Testosterone promotes dominance behaviors in the Ultimatum game after players’ status increases. Sci Rep. 2023;13(1):18029. 37865708 10.1038/s41598-023-45247-4 PMC10590433

[13] Kulikov AV, Osipova DV, Naumenko VS, Terenina E, Mormède P, Popova NK. A pharmacological evidence of positive association between mouse intermale aggression and brain serotonin metabolism. Behav Brain Res. 2012;233(1):113–9. 22561036 10.1016/j.bbr.2012.04.031

[14] Fodor A, Barsvari B, Aliczki M, Balogh Z, Zelena D, Goldberg SR, et al. The effects of vasopressin deficiency on aggression and impulsiveness in male and female rats. Psychoneuroendocrinology. 2014;47:141–50. 25001964 10.1016/j.psyneuen.2014.05.010

[15] Stribley JM, Carter CS. Developmental exposure to vasopressin increases aggression in adult prairie voles. Proc Natl Acad Sci U S A. 1999;96(22):12601–4. 10535968 10.1073/pnas.96.22.12601 PMC23008

[16] Ferris CF, Axelson JF, Martin AM, Roberge LF. Vasopressin immunoreactivity in the anterior hypothalamus is altered during the establishment of dominant/subordinate relationships between hamsters. Neuroscience. 1989;29(3):675–83. 2739905 10.1016/0306-4522(89)90140-1

[17] Delville Y, De Vries GJ, Schwartz WJ, Ferris CF. Flank-marking behavior and the neural distribution of vasopressin innervation in golden hamsters with suprachiasmatic lesions. Behav Neurosci. 1998;112(6):1486–501. 9926831 10.1037//0735-7044.112.6.1486

[18] Insel TR, Winslow JT. Central administration of oxytocin modulates the infant rat’s response to social isolation. Eur J Pharmacol. 1991;203(1):149–52. 1665788 10.1016/0014-2999(91)90806-2

[19] Pfundmair M, Reinelt A, DeWall CN, Feldmann L. Oxytocin strengthens the link between provocation and aggression among low anxiety people. Psychoneuroendocrinology. 2018;93:124–32. 29727809 10.1016/j.psyneuen.2018.04.025

[20] DeWall CN, Gillath O, Pressman SD, Black LL, Bartz JA, Moskovitz J, et al. When the love hormone leads to violence: Oxytocin increases intimate Partner Violence inclinations among High Trait Aggressive people. Soc Psychol Personal Sci. 2014;5(6):691–7.

[21] Gulevich R, Kozhemyakina R, Shikhevich S, Konoshenko M, Herbeck Y. Aggressive behavior and stress response after oxytocin administration in male Norway rats selected for different attitudes to humans. Physiol Behav. 2019;199:210–8. 30472394 10.1016/j.physbeh.2018.11.030

[22] Ramião E, Figueiredo P, Azeredo A, Moreira D, Barroso R, Barbosa F. Neurobiological characteristics of individuals who have committed sexual offenses: a systematic review. Aggress Violent Behav. 2023;72:101858.

[23] Tozdan S, Brunner F, Pietras L, Wiessner C, Briken P. Sexual aggression against males: differences between acts by males and females– results from the German Health and Sexuality Survey (GeSiD). Child Abuse Negl. 2021;117:105071. 33975258 10.1016/j.chiabu.2021.105071

[24] Jiwatram-Negrón T, Brooks MA, Ward M, Meinhart M. Systematic review of interventions to address suicidal behavior among people with a history of intimate partner violence: promises and gaps across the globe. Aggress Violent Behav. 2023;73:101871.

[25] Shors TJ, Tobόn K, DiFeo G, Durham DM, Chang HY. Sexual conspecific aggressive response (SCAR): a model of sexual trauma that disrupts maternal learning and plasticity in the female brain. Sci Rep. 2016;6:18960. 26804826 10.1038/srep18960 PMC4726239

[26] de Oliveira M, de Jong VE, Neumann TR. Modelling sexual violence in male rats: the sexual aggression test (SxAT). Transl Psychiatry. 2022;12(1):1–12. 35585046 10.1038/s41398-022-01973-3 PMC9117203

[27] Keloglan Musuroglu S, Ozturk DM, Sahin L, Cevik OS, Cevik K. Environmental enrichment as a strategy: attenuates the anxiety and memory impairment in social isolation stress. Int J Dev Neurosci. 2022;82(6):499–512. 35724417 10.1002/jdn.10205

[28] Ieraci A, Mallei A, Popoli M. Social isolation stress induces anxious-depressive-like Behavior and alterations of neuroplasticity-related genes in Adult Male mice. Neural Plast. 2016;2016:6212983. 26881124 10.1155/2016/6212983 PMC4736811

[29] Beiderbeck DI, Reber SO, Havasi A, Bredewold R, Veenema AH, Neumann ID. High and abnormal forms of aggression in rats with extremes in trait anxiety–involvement of the dopamine system in the nucleus accumbens. Psychoneuroendocrinology. 2012;37(12):1969–80. 22608548 10.1016/j.psyneuen.2012.04.011

[30] Koolhaas JM, Coppens CM, de Boer SF, Buwalda B, Meerlo P, Timmermans PJA. The resident-intruder paradigm: a standardized test for aggression, violence and social stress. J Vis Exp. 2013; (77): 4367. 10.3791/4367 PMC3731199 23852258

[31] Oliveira VE, de Lukas M, Wolf M, Durante HN, Lorenz E, Mayer A. AL, Oxytocin and vasopressin within the ventral and dorsal lateral septum modulate aggression in female rats. Nat Commun. 2021;12(1). 10.1038/s41467-021-23064-5 PMC8131389 34006875

[32] Braswell GS. An introduction to the Video Activity Coder: free software for coding videorecorded behaviors. Behav Res Methods. 2021;53(6):2596–603. 33970457 10.3758/s13428-021-01608-3

[33] Chari T, Griswold S, Andrews NA, Fagiolini M. The stage of the Estrus cycle is critical for interpretation of female mouse Social Interaction Behavior. Front Behav Neurosci 2020 Jun 30:14113. 10.3389/fnbeh.2020.00113 PMC7340104 32714163

[34] Lovick TA, Hélio Zangrossi J. Effect of Estrous Cycle on Behavior of females in Rodent tests of anxiety. Front Psychiatry 2021 Aug 31:12711065. 10.3389/fpsyt.2021.711065 PMC8438218 34531768

[35] Marcondes FK, Bianchi FJ, Tanno AP. Determination of the estrous cycle phases of rats: some helpful considerations. Braz J Biol. 2002;62(4 A):609–14. 12659010 10.1590/s1519-69842002000400008

[36] Singletart SJ, Kirsh AJ, WatsonJ, Karim BO, Huso DL, Hurn PD. Lack of correlation of vaginal impedance measurements with hormone levels in the rat. Contemp Top Lab Anim Sci Am Assoc Lab Anim Sci. 2005;44(6):37–42. PMC1403319 16370578

[37] Paccola CC, Resende CG, Stumpp T, Miraglia SM, Cipriano I. The rat estrous cycle revisited: a quantitative and qualitative analysis. Anim Reprod 2018 Jul 10(4):677–83.

[38] Carter CS. Postcopulatory sexual receptivity in the female hamster: the role of the ovary and adrenal. Horm Behav. 1972;3(3):261–5. 4681747 10.1016/0018-506x(72)90039-6

[39] Cora MC, Kooistra L, Travlos G. Vaginal cytology of the Laboratory Rat and mouse: review and criteria for the staging of the Estrous Cycle using stained vaginal smears. Toxicol Pathol. 2015;43(6):776–93. 25739587 10.1177/0192623315570339 PMC11504324

[40] Veening JG, Coolen LM. Neural mechanisms of sexual behavior in the male rat: emphasis on ejaculation-related circuits. Pharmacol Biochem Behav. 2014;121:170–83. 24368305 10.1016/j.pbb.2013.12.017

[41] Walf AA, Frye CA. The use of the elevated plus maze as an assay of anxiety-related behavior in rodents. Nat Protoc. 2007;2(2):322–8. 17406592 10.1038/nprot.2007.44 PMC3623971

[42] Seibenhener ML, Wooten MC. Use of the open field maze to measure locomotor and anxiety-like behavior in mice. J Vis Exp. 2015; (96): 52434. 10.3791/52434 PMC4354627 25742564

[43] Sturman O, Germain PL, Bohacek J. Exploratory rearing: a context- and stress-sensitive behavior recorded in the open-field test. Stress. 2018;21(5):443–52. 29451062 10.1080/10253890.2018.1438405

[44] La-Vu M, Tobias BC, Schuette PJ, Adhikari A. To Approach or avoid: an introductory overview of the study of anxiety using rodent assays. Front Behav Neurosci. 2020 Aug;26:14145. 10.3389/fnbeh.2020.00145 PMC7479238 33005134

[45] Aranda PS, Lajoie DM, Jorcyk CL. Bleach gel: a simple agarose gel for analyzing RNA quality. Electrophoresis. 2012;33(2):366. 22222980 10.1002/elps.201100335 PMC3699176

[46] Sikand K, Singh J, Ebron JS, Shukla GC. Housekeeping Gene Selection Advisory: Glyceraldehyde-3-Phosphate dehydrogenase (GAPDH) and β-Actin are targets of miR-644a. PLoS ONE. 2012;7(10):e47510. 23091630 10.1371/journal.pone.0047510 PMC3472982

[47] R Core Team. (2023). R: A Language and Environment for Statistical Computing_. R Foundation for Statistical Computing, Vienna, Austria. https://www.R-project.org/

[48] Bates D, Mächler M, Bolker B, Walker S. Fitting Linear mixed-effects models using lme4. J Stat Softw. 2015;67:1–48.

[49] Kuznetsova A, Brockhoff PB, Christensen RHB. lmerTest Package: tests in Linear mixed effects models. J Stat Softw. 2017;82:1–26.

[50] McCormick CM, Hodges TE, Simone JJ. Peer pressures: social instability stress in adolescence and social deficits in adulthood in a rodent model. Dev Cogn Neurosci. 2014;11:2–11. 24830945 10.1016/j.dcn.2014.04.002 PMC6989754

[51] Fone KCF, Porkess MV. Behavioural and neurochemical effects of post-weaning social isolation in rodents-relevance to developmental neuropsychiatric disorders. Neurosci Biobehav Rev. 2008;32(6):1087–102. 18423591 10.1016/j.neubiorev.2008.03.003

[52] Grippo AJ, Cushing BS, Carter CS. Depression-like behavior and stressor-induced neuroendocrine activation in female prairie voles exposed to chronic social isolation. Psychosom Med. 2007;69(2):149–57. 17289829 10.1097/PSY.0b013e31802f054b PMC3006075

[53] Oliveira VEM, Neumann ID, de Jong TR. Post-weaning social isolation exacerbates aggression in both sexes and affects the vasopressin and oxytocin system in a sex-specific manner. Neuropharmacology. 2019;156:107504. 30664846 10.1016/j.neuropharm.2019.01.019

[54] Wei S, Ji XW, Wu CL, Li ZF, Sun P, Wang JQ, et al. Resident intruder paradigm-induced aggression relieves depressive-like behaviors in male rats subjected to chronic mild stress. Med Sci Monit. 2014 Jun;9:20945–52. 10.12659/MSM.890200 PMC4067422 24911067

[55] Wongwitdecha N, Marsden CA. Social isolation increases aggressive behaviour and alters the effects of diazepam in the rat social interaction test. Behav Brain Res. 1996;75(1–2):27–32. 8800657 10.1016/0166-4328(96)00181-7

[56] Karin O, Raz M, Tendler A, Bar A, Korem Kohanim Y, Milo T, et al. A new model for the HPA axis explains dysregulation of stress hormones on the timescale of weeks. Mol Syst Biol. 2020;16(7):e9510. 32672906 10.15252/msb.20209510 PMC7364861

[57] Kinlein SA, Wilson CD, Karatsoreos IN. Dysregulated hypothalamic–pituitary–adrenal Axis function contributes to altered endocrine and neurobehavioral responses to acute stress. Front Psychiatry 2015 Mar 13:6:31. 10.3389/fpsyt.2015.00031 PMC4358064 25821436

[58] Ma Xcang, Jiang D, Jiang W, hui, Wang F, Jia M, Wu J, et al. Social isolation-induced aggression potentiates anxiety and depressive-like behavior in male mice subjected to unpredictable chronic mild stress. PLoS ONE. 2011;6(6):e20955. 21698062 10.1371/journal.pone.0020955 PMC3117867

[59] Mumtaz F, Khan MI, Zubair M, Dehpour AR. Neurobiology and consequences of social isolation stress in animal model—A comprehensive review. Biomed Pharmacother. 2018;Sep:105:1205–22. 10.1016/j.biopha.2018.05.086 30021357

[60] Veenema AH. Early life stress, the development of aggression and neuroendocrine and neurobiological correlates: what can we learn from animal models? Front Neuroendocrinol. 2009;30(4):497–518. 19341763 10.1016/j.yfrne.2009.03.003

[61] Neumann ID, Veenema AH, Beiderbeck DI. Aggression and anxiety: Social Context and Neurobiological Links. Front Behav Neurosci. 2010;4:12. 20407578 10.3389/fnbeh.2010.00012 PMC2854527

[62] Guo L, Qi YJ, Tan H, Dai D, Balesar R, Sluiter A, et al. Different oxytocin and corticotropin-releasing hormone system changes in bipolar disorder and major depressive disorder patients. eBioMedicine. 2022;84:104266. 36126617 10.1016/j.ebiom.2022.104266 PMC9489957

[63] Kim J, Park Y, Kim EJ, Jung H, Yoon M. Relationship between oxytocin and serotonin and the fearfulness, dominance, and trainability of horses. J Anim Sci Technol. 2021;63(2):453–60. 33987618 10.5187/jast.2021.e29 PMC8071747

[64] Kawada A, Nagasawa M, Murata A, Mogi K, Watanabe K, Kikusui T, et al. Vasopressin enhances human preemptive strike in both males and females. Sci Rep. 2019;9(1):9664. 31273244 10.1038/s41598-019-45953-y PMC6609689

[65] MacLean EL, Gesquiere LR, Gruen ME, Sherman BL, Martin WL, Carter CS. Endogenous oxytocin, Vasopressin, and aggression in domestic dogs. Front Psychol. 2017;8:1613. 29021768 10.3389/fpsyg.2017.01613 PMC5624304

[66] Ferris CF, Albers HE, Wesolowski SM, Goldman BD, Luman SE. Vasopressin injected into the hypothalamus triggers a stereotypic behavior in golden hamsters. Science. 1984;224(4648):521–3. 6538700 10.1126/science.6538700

[67] Albers HE, Ferris CF. Role of the flank gland in vasopressin induced scent marking behavior in the hamster. Brain Res Bull. 1986;17(3):387–9. 3768740 10.1016/0361-9230(86)90242-x

[68] Politch JA, Leshner AI. Relationship between plasma corticosterone levels and levels of aggressiveness in mice. Physiol Behav. 1977;19(6):775–80. 204951 10.1016/0031-9384(77)90314-6

[69] Veenit V, Cordero MI, Tzanoulinou S, Sandi C. Increased corticosterone in peripubertal rats leads to long-lasting alterations in social exploration and aggression. Front Behav Neurosci. 2013;7:26. 23576965 10.3389/fnbeh.2013.00026 PMC3616252

[70] Witte AV, Flöel A, Stein P, Savli M, Mien LK, Wadsak W, et al. Aggression is related to frontal serotonin-1A receptor distribution as revealed by PET in healthy subjects. Hum Brain Mapp. 2009;30(8):2558–70. 19086022 10.1002/hbm.20687 PMC6870783

[71] Kästner N, Richter SH, Urbanik S, Kunert J, Waider J, Lesch KP, et al. Brain serotonin deficiency affects female aggression. Sci Rep. 2019;9:1366. 30718564 10.1038/s41598-018-37613-4 PMC6361930

[72] Neumann ID, Landgraf R. Balance of brain oxytocin and vasopressin: implications for anxiety, depression, and social behaviors. Trends Neurosci. 2012;35(11):649–59. 22974560 10.1016/j.tins.2012.08.004

[73] Veenema AH, Neumann ID. Central vasopressin and oxytocin release: regulation of complex social behaviours. Prog Brain Res. 2008;170:261–76. 18655888 10.1016/S0079-6123(08)00422-6

[74] Ferris CF Jr, Koppel RHM, Perry G, Fuller KW, Delville RW. Vasopressin/Serotonin interactions in the Anterior Hypothalamus Control Aggressive Behavior in Golden hamsters. J Neurosci. 1997;17(11):4331–40. 9151749 10.1523/JNEUROSCI.17-11-04331.1997 PMC6573530

[75] Charles R, Sakurai T, Takahashi N, Elder GA, Gama Sosa MA, Young LJ, et al. Introduction of the human AVPR1A gene substantially alters brain receptor expression patterns and enhances aspects of social behavior in transgenic mice. Dis Model Mech. 2014;7(8):1013–22. 24924430 10.1242/dmm.017053 PMC4107330

[76] Wersinger SR, Caldwell HK, Martinez L, Gold P, Hu SB. Young 3rd WS. Vasopressin 1a receptor knockout mice have a subtle olfactory deficit but normal aggression. Genes Brain Behav. 2007;6(6):540–51. 17083331 10.1111/j.1601-183X.2006.00281.x

[77] Rigney N, de Vries GJ, Petrulis A. Modulation of social behavior by distinct vasopressin sources. Front Endocrinol. 2023;14:1127792. 10.3389/fendo.2023.1127792 PMC9968743 36860367

[78] Friedman NP, Robbins TW. The role of prefrontal cortex in cognitive control and executive function. Neuropsychopharmacology. 2022;47(1):72–89. 34408280 10.1038/s41386-021-01132-0 PMC8617292

[79] Kenyon AR, Alvares GA, Hickie IB, Guastella AJ. The effects of Acute Arginine Vasopressin Administration on Social Cognition in Healthy Males. J Horm. 2013;2013:e386306.

[80] Patel TN, Caiola HO, Mallari OG, Blandino KL, Goldenthal AR, Dymecki SM, et al. Social interactions increase activation of vasopressin-responsive neurons in the dorsal raphe. Neuroscience. 2022;495:25–46. 35654294 10.1016/j.neuroscience.2022.05.032

[81] van Erp AMM, Miczek KA, Aggressive Behavior. Increased Accumbal dopamine, and decreased cortical serotonin in rats. J Neurosci. 2000;20(24):9320–5. 11125011 10.1523/JNEUROSCI.20-24-09320.2000 PMC6773005

[82] Ukkola-Vuoti L, Oikkonen J, Onkamo P, Karma K, Raijas P, Järvelä I. Association of the arginine vasopressin receptor 1A (AVPR1A) haplotypes with listening to music. J Hum Genet. 2011;56(4):324–9. 21307861 10.1038/jhg.2011.13

[83] Rae M, Duarte ML, Gomes I, Camarini R, Devi LA. Oxytocin and vasopressin: signalling, behavioural modulation and potential therapeutic effects. Br J Pharmacol. 2022;179(8):1544–64. 33817785 10.1111/bph.15481 PMC8488062

[84] Cid-Jofré V, Moreno M, Reyes-Parada M, Renard GM. Role of Oxytocin and Vasopressin in Neuropsychiatric disorders: therapeutic potential of agonists and antagonists. Int J Mol Sci. 2021;22(21):12077. 34769501 10.3390/ijms222112077 PMC8584779

[85] Cilz NI, Cymerblit-Sabba A, Young WS. Oxytocin and vasopressin in the rodent hippocampus. Genes Brain Behav. 2019;18(1):e12535. 30378258 10.1111/gbb.12535

[86] Jones C, Barrera I, Brothers S, Ring R, Wahlestedt C. Oxytocin and social functioning. Dialogues Clin Neurosci. 2017;19(2):193. 28867943 10.31887/DCNS.2017.19.2/cjones PMC5573563

[87] Kubzansky LD, Mendes WB, Appleton AA, Block J, Adler GK. A heartfelt response: Oxytocin effects on response to social stress in men and women. Biol Psychol. 2012;90(1):1–9. 22387929 10.1016/j.biopsycho.2012.02.010 PMC3327158

[88] Takayanagi Y, Onaka T. Roles of oxytocin in stress responses, Allostasis and Resilience. Int J Mol Sci. 2022;23(1):150. 10.3390/ijms23010150 PMC8745417 35008574

[89] Winslow JT, Insel TR. Social status in pairs of male squirrel monkeys determines the behavioral response to central oxytocin administration. J Neurosci off J Soc Neurosci. 1991;11(7):2032–8. 10.1523/JNEUROSCI.11-07-02032.1991 PMC6575463 1648603

[90] Gobrogge KL, Liu Y, Young LJ, Wang Z. Anterior hypothalamic vasopressin regulates pair-bonding and drug-induced aggression in a monogamous rodent. Proc Natl Acad Sci. 2009;106(45):19144–9. 19858480 10.1073/pnas.0908620106 PMC2776424

[91] Coccaro EF, Kavoussi RJ, Hauger RL, Cooper TB, Ferris CF. Cerebrospinal fluid vasopressin levels: correlates with aggression and serotonin function in personality-disordered subjects. Arch Gen Psychiatry. 1998;55(8):708–14. 9707381 10.1001/archpsyc.55.8.708

[92] Haller J, Fuchs E, Halász J, Makara GB. Defeat is a major stressor in males while social instability is stressful mainly in females: towards the development of a social stress model in female rats. Brain Res Bull. 1999;50(1):33–9. 10507469 10.1016/s0361-9230(99)00087-8

[93] Summers CH, Winberg S. Interactions between the neural regulation of stress and aggression. J Exp Biol. 2006;209(Pt 23):4581–9. 17114393 10.1242/jeb.02565

[94] Jennings KJ, de Lecea L. Neural and hormonal control of sexual behavior. Endocrinology. 2020;161(10):1–13. 10.1210/endocr/bqaa150 PMC7507403 32845294

[95] Rodríguez-Nieto G, Sack AT, Dewitte M, Emmerling F, Schuhmann T. The Modulatory Role of Cortisol in the regulation of sexual behavior in Young males. Front Behav Neurosci. 2020;14:552567. 33250723 10.3389/fnbeh.2020.552567 PMC7674834

[96] Retana-Marquez S, Bonilla-Jaime H, Velazquez-Moctezuma J. Lack of Effect of Corticosterone Administration on male sexual behavior of rats. Physiol Behav. 1998;63(3):367–70. 9469728 10.1016/s0031-9384(97)00437-x

[97] Popova NK, Tsybko AS, Naumenko VS. The implication of 5-HT receptor family members in aggression, depression and suicide: similarity and difference. Int J Mol Sci. 2022;23(15):8814. 35955946 10.3390/ijms23158814 PMC9369404

[98] de Boer SF, Koolhaas JM. 5-HT1A and 5-HT1B receptor agonists and aggression: a pharmacological challenge of the serotonin deficiency hypothesis. Eur J Pharmacol. 2005;526(1–3):125–39. 16310183 10.1016/j.ejphar.2005.09.065

[99] Higley JD, Mehlman PT, Poland RE, Taub DM, Vickers J, Suomi SJ, et al. CSF testosterone and 5-HIAA correlate with different types of aggressive behaviors. Biol Psychiatry. 1996;40(11):1067–82. 8931909 10.1016/S0006-3223(95)00675-3

[100] Tan O, Musullulu H, Raymond JS, Wilson B, Langguth M, Bowen MT. Oxytocin and vasopressin inhibit hyper-aggressive behaviour in socially isolated mice. Neuropharmacology. 2019;156. 10.1016/j.neuropharm.2019.03.016 30885607

[101] Heinrichs M, von Dawans B, Domes G. Oxytocin, vasopressin, and human social behavior. Front Neuroendocrinol. 2009;30(4):548–57. 19505497 10.1016/j.yfrne.2009.05.005

[102] Blanchard RJ, McKittrick CR, Blanchard DC. Animal models of social stress: effects on behavior and brain neurochemical systems. Physiol Behav. 2001;73(3):261–71. 11438351 10.1016/s0031-9384(01)00449-8

[103] Dimonte S, Sikora V, Bove M, Morgese MG, Tucci P, Schiavone S, et al. Social isolation from early life induces anxiety-like behaviors in adult rats: relation to neuroendocrine and neurochemical dysfunctions. Biomed Pharmacother. 2023;158:114181. 36592494 10.1016/j.biopha.2022.114181

[104] Grigoryan GA, Pavlova IV, Zaichenko MI. Effects of Social isolation on the development of anxiety and Depression-Like Behavior in Model experiments in animals. Neurosci Behav Physiol. 2022;52(5):722–38. 36119650 10.1007/s11055-022-01297-1 PMC9471030

[105] Eraslan E, Castelhano-Carlos MJ, Amorim L, Soares-Cunha C, Rodrigues AJ, Sousa N. Home-cage behavior is impacted by stress exposure in rats. Front Behav Neurosci. 2023;17:1195011. 37358966 10.3389/fnbeh.2023.1195011 PMC10288110

[106] Brandt L, Liu S, Heim C, Heinz A. The effects of social isolation stress and discrimination on mental health. Transl Psychiatry. 2022;12:398. 36130935 10.1038/s41398-022-02178-4 PMC9490697

[107] Loades ME, Chatburn E, Higson-Sweeney N, Reynolds S, Shafran R, Brigden A, et al. Rapid systematic review: the impact of social isolation and loneliness on the Mental Health of Children and adolescents in the Context of COVID-19. J Am Acad Child Adolesc Psychiatry. 2020;59(11):1218–e12393. 32504808 10.1016/j.jaac.2020.05.009 PMC7267797

[108] Dayan P, Huys QJM. Serotonin, inhibition, and negative Mood. PLoS Comput Biol. 2008;4(2):e4. 18248087 10.1371/journal.pcbi.0040004 PMC2222921

[109] Siegel JZ, Crockett MJ. How serotonin shapes moral judgment and behavior. Ann N Y Acad Sci. 2013;1299(1):42–51. 25627116 10.1111/nyas.12229 PMC3817523

[110] Csikota P, Fodor A, Balázsfi D, Pintér O, Mizukami H, Weger S, et al. Vasopressinergic control of stress-related behavior: studies in Brattleboro rats. Stress Amst Neth. 2016;19(4):349–61. 10.1080/10253890.2016.1183117 27187740

[111] Fodor A, Kovács KB, Balázsfi D, Klausz B, Pintér O, Demeter K, et al. Depressive- and anxiety-like behaviors and stress-related neuronal activation in vasopressin-deficient female Brattleboro rats. Physiol Behav. 2016;158:100–11. 26939727 10.1016/j.physbeh.2016.02.041

[112] Kinlein SA, Phillips DJ, Keller CR, Karatsoreos IN. Role of corticosterone in altered neurobehavioral responses to acute stress in a model of compromised hypothalamic-pituitary-adrenal axis function. Psychoneuroendocrinology. 2019;102:248–55. 30594817 10.1016/j.psyneuen.2018.12.010 PMC7649055

[113] Lam VY, Raineki C, Wang LY, Chiu M, Lee G, Ellis L, et al. Role of corticosterone in anxiety- and depressive-like behavior and HPA regulation following prenatal alcohol exposure. Prog Neuropsychopharmacol Biol Psychiatry. 2019;90:1–15. 30367959 10.1016/j.pnpbp.2018.10.008 PMC6449057

[114] Zhang F, Dou J, Zhao X, Luo H, Ma L, Wang L, et al. Identification of key genes Associated with heat stress in rats by Weighted Gene Co-expression Network Analysis. Anim Open Access J MDPI. 2023;13(10):1618. 10.3390/ani13101618 PMC10215599 37238049

[115] Rao MS, Van Vleet TR, Ciurlionis R, Buck WR, Mittelstadt SW, Blomme EAG, et al. Comparison of RNA-Seq and microarray gene expression platforms for the Toxicogenomic evaluation of liver from short-term rat toxicity studies. Front Genet. 2018;9:636. 30723492 10.3389/fgene.2018.00636 PMC6349826

[116] Silver N, Cotroneo E, Proctor G, Osailan S, Paterson KL, Carpenter GH. Selection of housekeeping genes for gene expression studies in the adult rat submandibular gland under normal, inflamed, atrophic and regenerative states. BMC Mol Biol. 2008;9:64. 18637167 10.1186/1471-2199-9-64 PMC2492873

[117] Shalaginova IG, Tuchina OP, Turkin AV, Vylegzhanina AE, Nagumanova AN, Zachepilo TG, et al. The effect of long-term emotional and painful stress on the expression of Proinflammatory Cytokine genes in rats with high and low excitability of the nervous system. J Evol Biochem Physiol. 2023;59(2):642–52. 37128572 10.1134/S0022093023020291 PMC10132918

[118] Krause EG, de Kloet AD, Flak JN, Smeltzer MD, Solomon MB, Evanson NK, et al. Hydration state controls stress responsiveness and social behavior. J Neurosci off J Soc Neurosci. 2011;31(14):5470–6. 10.1523/JNEUROSCI.6078-10.2011 PMC3086063 21471383

[119] Berends YR, Tulen JHM, Wierdsma AI, Van Pelt J, Feldman R, Zagoory-Sharon O, et al. Oxytocin and vasopressin in male forensic psychiatric patients with personality disorders and healthy controls. J Forensic Psychiatry Psychol. 2022;33(1):130–51.

[120] Edem EE, Oguntala OA, Ikuelogbon DA, Nebo KE, Fafure AA, Akinluyi ET, et al. Prolonged ketamine therapy differentially rescues psychobehavioural deficits via modulation of nitro-oxidative stress and oxytocin receptors in the gut-brain-axis of chronically-stressed mice. Psychoneuroendocrinology. 2023;158:106370. 37678086 10.1016/j.psyneuen.2023.106370

[121] Dubovicky M, Mach M, Key M, Morris M, Paton S, Lucot JB. Diurnal behavioral and endocrine effects of chronic shaker stress in mice. Neuro Endocrinol Lett. 2007;28(6):846–53. 18063939

