# Supplementary material for: Social isolation induces sexually aggressive behaviour in male Wistar rats
Source: BMC Neurosci. 2025 Feb 26;26:15. doi: 10.1186/s12868-025-00932-0 (PMC11866782; doi:10.1186/s12868-025-00932-0)
Supplement: Supplementary file 1 — Supplementary Material 1 [file 12868_2025_932_MOESM1_ESM.docx]

**Social isolation induces sexually aggressive behaviour in male Wistar rats: Supplementary material**

Supplementary Table 1: Genes investigated using TaqMan® gene expression assays.

| Probes | Gene symbol | Assay ID |
| --- | --- | --- |
| Oxytocin receptor | OXTR | Rn00563503_m1 |
| Arginine vasopressin receptor type 1 | AVPR1A | Rn00583910_m1 |
| Corticotropin releasing hormone receptor 1 | CRHR1 | Rn01463997_m1 |
| Androgen receptor | AR | Rn00664479_m1 |
| 5-hydroxytryptamine (serotonin) receptor 1A | HTR1A | Rn00561409_s1 |
| Glyceraldehyde-3-phosphate dehydrogenase | GAPDH | Rn01775763_g1 |

Supplementary table 2: Neurochemical marker concentrations according to experimental group

| Marker | GHCM | GHSM | ICM | ISM | Group housing | Isolation housing |
| --- | --- | --- | --- | --- | --- | --- |
| Testosterone (ng/ml) ^@^ | 4.48  (1.95 - 5.24) | 2.37  (1.37 - 2.82) | 1.87  (1.63 - 3.61) | 2.82  (2.59 - 3.80) | 2.42  (1.74 - 4.19) | 2.59  (1.77 - 4.07) |
| Serotonin (ng/ml) ^#^ | 694.24 ± 115.61 | 1018.85 ± 129.83 | 448.32 ± 94.93 | 825.10 ± 94.31 | 856.55 ± 205.71 | 636.71 ± 215.60 |
| CRH (ng/ml) ^@^ | 23.24  (22.36 - 24.53) | 16.10  (13.75 - 18.95) | 16.32  (12.55 - 18.20) | 17.55  (14.85 - 18.15) | 20.41  (16.55 - 23.08) | 16.59  (13.84 - 18.30) |
| Corticosterone (ng/ml) ^@^ | 22.34  (18.65 - 24.78) | 19.81  (16.09 - 20.00) | 35.77  (31.89 - 47.86) | 22.79  (20.46 - 24.33) | 19.82  (16.60 - 22.62) | 31.12  (22.85 - 34.85) |
| Oxytocin (pg/ml) ^@^ | 676.00  (620.80 - 825.30) | 614.70  (593.05 - 714.20) | 235.75  (186.48 - 259.50) | 306.25  (251.35 - 319.38) | 661.90  (597.10 - 728.69) | 254.48  (215.12 - 300.21) |
| AVP (pg/ml) ^#^ | 2.26 ± 0.49 | 2.55 ± 0.66 | 4.40 ± 0.63 | 4.84 ± 0.82 | 2.40 ± 0.58 | 4.62 ± 0.74 |

Group housing reflects GHCM and GHSM rats. Isolation housing reflects ICM and ISM rats. ^#^ Normally distributed data are reported as mean ± standard deviation. ^@^ Non-parametrically distributed data are reported as median and 25^th^ and 75 percentiles. AVP = arginine vasopressin, CRH = corticotropic-releasing hormone, GHCM = group housed control males, GHSM = group housed sexual aggression assessment males, ICM = isolated control males, ISM = isolated sexual aggression assessment males
